# Community-level interventions for mitigating the risk of waterborne diarrheal diseases: a systematic review

**DOI:** 10.1186/s13643-022-01947-y

**Published:** 2022-04-18

**Authors:** Chisala D. Meki, Esper J. Ncube, Kuku Voyi

**Affiliations:** 1 University of Zambia, School of Public Health, University of Zambia, P O. BOX 50110, Lusaka, Zambia; 2 School of Health Systems and Public Health, University of Pretoria, Pretoria, South Africa; 3 Rand Water, Johannesburg, South Africa

## Abstract

**Background:**

Waterborne diarrhea diseases are among the leading causes of morbidity and mortality globally. These diseases can be mitigated by implementing various interventions. We reviewed the literature to identify available interventions to mitigate the risk of waterborne diarrheal diseases.

**Methods:**

We conducted a systematic database review of CINAHL (Cumulative Index to Nursing and Allied Health Literature), PubMed, Web of Science Core Collection, Cochrane library, Scopus, African Index Medicus (AIM), and LILACS (Latin American and Caribbean Health Sciences Literature). Our search was limited to articles published between 2009 and 2020. We conducted the review using the Preferred Reporting Items for Systematic Reviews and Meta-Analyses (PRISMA) statement checklist. The identified studies were qualitatively synthesized.

**Results:**

Our initial search returned 28 773 articles of which 56 studies met the inclusion criteria. The included studies reported interventions, including vaccines for rotavirus disease (monovalent, pentavalent, and Lanzhou lamb vaccine); enhanced water filtration for preventing cryptosporidiosis, Vi polysaccharide for typhoid; cholera 2-dose vaccines, water supply, water treatment and safe storage, household disinfection, and hygiene promotion for controlling cholera outbreaks.

**Conclusion:**

We retrieved few studies on interventions against waterborne diarrheal diseases in low-income countries. Interventions must be specific to each type of waterborne diarrheal disease to be effective. Stakeholders must ensure collaboration in providing and implementing multiple interventions for the best outcomes.

**Systematic review registration:**

PROSPERO CRD42020190411.

**Supplementary Information:**

The online version contains supplementary material available at 10.1186/s13643-022-01947-y.

## Background

Waterborne diseases are transmitted through drinking water that is contaminated with human or animal fecal matter containing pathogenic microorganisms [1], including viruses, bacteria, and protozoa that survive and multiply in food, water, and other surfaces [2, 3]. Most waterborne diseases including cholera, dysentery (shigellosis and amebiasis), typhoid, cryptosporidiosis, giardiasis, cyclosporiasis, yersiniosis, salmonellosis, campylobacteriosis, and other gastroenteritis infections caused by rotavirus, adenovirus norovirus, enterovirus, caliciviruses, astroviruses, and reoviruses manifest as diarrhea [4]. Diarrhea is one of the major causes of mortality and morbidity around the world especially among children [5–8].

Morbidity and mortality from diarrheal diseases can be reduced by applying various interventions that help to cut the fecal-oral transmission route. These interventions include providing adequate and safe water, proper sanitation, handwashing facilities, practicing personal hygiene and food hygiene, education, and vaccinations [9–14]. Exclusive breastfeeding has also been shown to reduce infant morbidity and mortality from diarrheal diseases [15–17].

The risk of diarrhea can be reduced by washing hands (48%), improving water quality (17%), and disposing of excreta properly (36%) [18]. At a global scale, proper water sanitation and hygiene may reduce the global diarrheal disease burden by 9.1% and reduce mortality by 6.3% [19]. Despite water, sanitation, and hygiene being critical in preventing and controlling diarrheal diseases, only 71% of people globally have access to safely managed water sources, 45% of people have access to adequate safely managed sanitation, and 60% of people have access to basic handwashing facilities [20].

The different interventions available to prevent and control diarrheal diseases on a global scale have been reviewed and summarized previously. Previous reviews focused on the importance of proper excreta management in preventing diarrhea diseases [21], improving water quality for preventing diarrhea [22], house fly control to prevent diarrhea [23], handwashing to prevent diarrhea [24], a review of the rotavirus vaccine [25], and a review on vaccines for preventing cholera, shigella, enterotoxigenic *Escherichia coli* (ETEC), and rotavirus [10]. In this review, we limited our search to the most recent studies, those published between 2009 and 2020. This will provide an updated review of available interventions against waterborne pathogens that cause diarrhea. Instead of reviewing interventions to reduce the risk of diarrhea in general, we reviewed interventions to mitigate the risk of waterborne diarrhea diseases at the community level. Our review focusses on diseases caused by pathogens that are found in water contaminated by human or animal excreta. These diseases include cholera, dysentery (shigellosis and amebiasis), typhoid, cryptosporidiosis, giardiasis, cyclosporiasis, yersiniosis, salmonellosis, campylobacteriosis, and other gastroenteritis infections caused by rotavirus, adenovirus norovirus, enterovirus, caliciviruses, astroviruses, and reoviruses.

Systematic reviews are critical to informing evidence-based policy. This review may help to formulate new policies to mitigate the risk of waterborne diarrhea diseases. Mitigating the risk of waterborne diarrhea disease is vital to achieving the Sustainable Development Goal (SDG) goal number six (SGD-6), ensuring access to water and sanitation for all. To achieve this goal, it is vital that we know which interventions are available to mitigate the risk of waterborne diarrheal disease, globally.

### Review question

Which community levels interventions exist to mitigate the risk of waterborne diarrheal diseases?

## Methods

### Protocol and registration

The review was conducted between May 2020 and February 2022. The review protocol was registered on University of York Prospero registration number CRD42020190411. The protocol is currently available on CRD42020190411.

### Eligibility criteria

We only included studies published from 2009 to 2020 to ensure the inclusion of current information. We only included studies published in English due to a lack of resources for translation. We included studies conducted from across the globe.

### Participants/population

We reviewed studies that included participants of all ages from all communities affected by waterborne diarrheal diseases. Our review focused on intervention(s) of interest including water supply, sanitation, hygiene including handwashing, health promotion and education, vaccinations, and breastfeeding. We did not include a specific comparison/control group in this review and included studies without control groups.

### Inclusion criteria

We included studies of any design that had complete methods, results, and discussion sections. To avoid duplication of results, we excluded other reviews. We also excluded documents that summarized other studies such as letters to editors, comment papers, brief reports, abstracts, and research news. Table 1 presents the inclusion and exclusion criteria applied in this review.

**Table 1:** Inclusion and exclusion criteria used to select studies describing interventions for mitigating the risk of waterborne diarrheal diseases

**Inclusion criteria**	
1. Studies published from 2009 to 2020	
2. Full and complete studies	
3. Studies conducted across the world	
4. Studies conducted in real community-level settings i.e., schools, health facilities, and households in humans	
5. Studies with water supply, sanitation, hygiene, breastfeeding, and vaccination interventions	
6. Studies with a waterborne diarrheal disease outcome or water quality outcome	
7. Studies reporting effective intervention outcome	
8. Studies with confirmed uptake of an intervention(s)	
**Exclusion criteria**	
1. Studies not published in the English language	
2. Studies with waterborne diarrheal disease(s) and another disease(s) outcome	
3. Studies that the researcher did not have full-text access to.	
4. Studies that reported hospital-acquired infections/nosocomial infection	

### Condition studied

We reviewed studies of waterborne diarrheal diseases that are transmitted to humans when they consume water contaminated with pathogens of human or animal excreta. Specifically, waterborne diseases with a diarrheal outcome including cholera, dysentery (shigellosis and amebiasis), typhoid, cryptosporidiosis, giardiasis, cyclosporiasis, yersiniosis, salmonellosis campylobacteriosis, and other gastroenteritis infections caused by rotavirus, adenovirus norovirus, enterovirus, caliciviruses, astroviruses, and reoviruses.

### Effect measure

We reviewed studies that reported the following measures: frequencies, proportions, prevalence, odds ratios, rate ratio (incidence rate ratio), relative risk, period incidence, median/range, and risk of disease. Our review did not exclude studies based on predetermined measures of effects.

### Information about searches

We searched databases including CINAHL via EBSCOHOST, PubMed, Scopus, Cochrane Library, and Web of Science Core Collection. Databases were searched in CINAHL using major headings and free search; PubMed using title and abstract, mesh terms; Scopus using titles /abstract; Web of Science using topics and Cochrane library using title/abstract and Mesh terms. The search terms were identified with the help of a librarian. Synonyms for the keywords were identified and used in the search. Truncations were used to retrieve variants of keywords and Boolean operators ‘OR’ and ‘AND’ were also used to combine words for searching in each database. Full search strategies for each database are presented in Supplementary Table 1. In addition, African Index Medicus and LILACS databases were searched via the Global Index Medicus database on 10th February 2022 using title, abstract, and subject. The phrases used in the search included the following: “Waterborne” and “disease* OR infect* OR illness* OR outbreak* OR sickness*” and “cholera OR rotavirus* OR reovirus* OR shigell* OR enterovirus* OR calicivir* OR norovirus* OR astrovirus* OR adenovirus* OR giard* OR cyclosporia* OR dysenter* OR cryptosporid* OR yersin* OR salmonell* OR typhoid* OR campylobacter* OR amoebia*” and “intervention*.” Note that we did not find any studies that met the inclusion criteria after the two databases were searched.

### Data selection process

After the initial search, we downloaded all studies to Endnote reference manager. Firstly, we removed duplicate articles. Chisala D. Meki (CDM) and Esper J. Ncube (EJN) screened the titles and abstracts of all the articles. We excluded all articles that did not meet the inclusion criteria, and then read the remaining articles in detail. After the preliminary screening, CDM and EJN read the full-text articles, Kuku Voyi (KV) validated the screening and resolved any disputes. We extracted data from included studies using a data extraction form (Table 2) adapted from Cochrane, available online: https://www.cochranelibrary.com/cdsr/doi/10.1002/14651858.MR000044/full. The data collection sheet was piloted before use. CDM and EJN extracted data and KV resolved any disputes. We searched the reference lists of included articles to check for relevant studies, but we could not identify any new studies.

**Table 2:** Data extraction sheet used to extract information from studies describing interventions for mitigating the risk of waterborne diarrheal disease

Items	Comments
1. Title of study	
2. Authors details	
3. Year of publication	
4. Aim or objective(s) of the study	
5. Type of waterborne diarrheal disease(s)	
6. Study design	
7. Country setting (rural or urban or mixed)	
8. Type of community settings (school, household, health facility)	
9. Participants and cases	
10. Participant’s comparison or control group if available	
11. Sample size	
12. Types of intervention(s)	
13. Intervention comparison or control group if available	
14. Data analysis outcome measures	
15. Results and effects of the interventions	
16. Conclusion	
17. Funding	

### Data synthesis

Data were synthesized qualitatively, and results summarized narratively. We did not do a meta-analysis because the studies had different study designs, outcomes, participants, sample sizes, interventions, locations, contexts, and so on. Data were synthesized using thematic analysis and presented under different themes guided by the data extraction tool and findings.

### Risk of bias (quality) assessment

A librarian from the University of Pretoria, Faculty of Health Sciences was involved at all levels of the study to ensure quality. The librarian helped to formulate search strategies and identify the right search terms, keywords, and synonyms. The librarian helped to retrieve relevant literature. CDM and EJN created the inclusion and exclusion criteria of the search, selected the literature, assessed quality, and analyzed data. KV helped to resolve disagreements between the two reviewers. We checked the quality of each study included in this review using checklists from Joanna Briggs Institute (JBI) University of Adelaide and the strobe checklist for observational studies at the point of data extraction. This was done to ensure that all the included studies reported the elements required to assess the quality of studies. We did not assess the risk of bias across studies because this was not a meta-analysis.

## Results

### Study selection

**Fig. 1:**
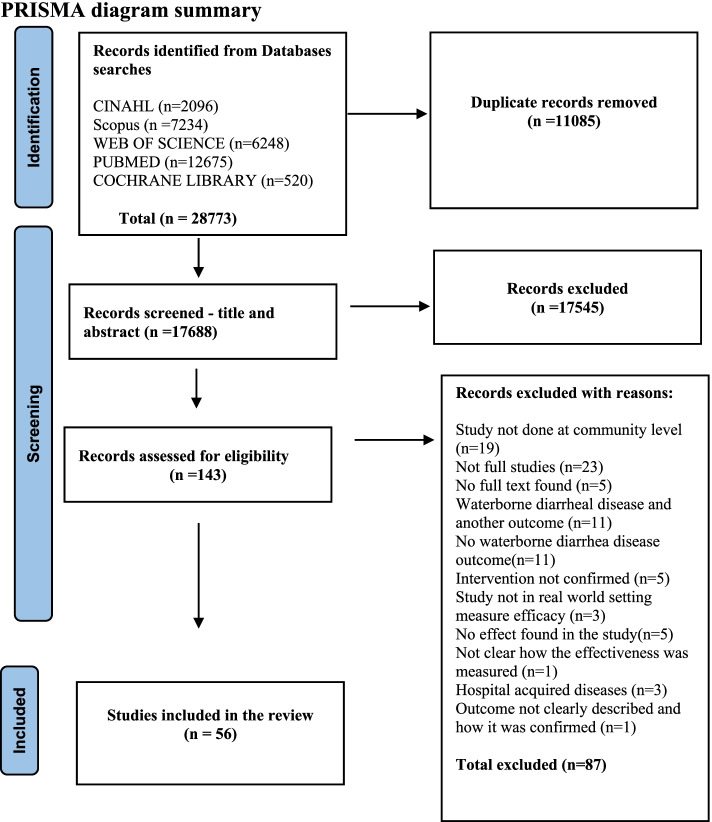
PRISMA diagram on selecting studies describing interventions for mitigating the risk of waterborne diarrheal disease

The initial search returned 28773 studies. After removing duplicates, we screened the titles and abstracts of 17688 articles, of which 143 were screened in full. Eighty-seven articles were excluded after full screening. After full screening, 56 studies that met the inclusion criteria were finally included in the review. The details for the selection of studies are presented in the PRISMA diagram (Fig. 1). A full list of included and excluded studies with reasons is presented in (Supplementary Appendix 1).

### Characteristics of studies

#### Type of waterborne diarrheal diseases

Most of the studies done at the community level reported interventions against rotavirus diseases (*n* = 49), five studies reported interventions against cholera, and one study looked at typhoid and another cryptosporidiosis (Supplementary Table 2). We could not find any studies that met the inclusion criteria describing interventions against dysentery (shigellosis and amebiasis), giardiasis, cyclosporiasis, yersiniosis, salmonellosis, campylobacteriosis, and other gastroenteritis infections caused by adenovirus norovirus, enterovirus, caliciviruses, astroviruses, and reoviruses.

#### Study settings

We selected studies conducted at the community level, most of these studies were conducted in healthcare facilities (*n* = 51), followed by households and other community settings (*n* = 5) (Supplementary Table 2).

#### Study designs

We reviewed 22 case-control studies and 13 studies that were combined surveillance and case-control studies. Nine of the included studies were surveillance studies and three were cohort studies. The rest of the included studies were a preliminary community trial (*n* = 1), cluster-randomized control trial (*n* = 1), cluster-randomized effectiveness trial study (*n* = 1), case study (*n* = 1), retrospective observational study (*n* = 1), retrospective database study (*n* = 1), combined case-control and cohort study (*n* = 1), retrospective analysis (*n* = 1), and a combined time series and case-control study (*n* = 1) (Supplementary Table 2).

#### Countries and economic status of the included studies

The included studies were conducted in 37 countries, with most studies from the United States (*n* = 7), three conducted in China, and the rest from a variety of other countries. According to the World Bank economic classification, most of the studies were conducted in high-income countries (*n* = 24), followed by lower middle-income countries (*n* = 15) then upper middle-income (*n* = 11), and six studies in low-income countries (Supplementary Table 2).

#### Age groups of study participants

Included studies included participants of different age categories. Most of the studies looked at interventions to mitigate the risk of waterborne diarrheal diseases in children younger than 5 years old (*n* = 47), children and adults (*n* = 6), children younger than 16 years (*n* = 1), older than 12 years (*n* = 1) and another study involved children younger than 8 years old (Supplementary Table 3).

### Types of interventions

Many studies (*n* = 49) looked at the rotavirus vaccine of which 22 reported the rotavirus monovalent (RV1) Rotarix vaccine; nine studies investigated the pentavalent (R5) Rotateq vaccine; 16 studies investigated Rotarix and Rotateq vaccine; and two other studies addressed Lanzhou lamb rotavirus vaccines. One study considered emergency water supply, household water treatment and safe storage, home disinfection, and hygiene promotion at the community level. Four (4) studies reported the 2-dose oral cholera vaccine. Another study reported water treatment through enhanced filtration and another study reported Vi polysaccharide vaccination (Supplementary Table 3).

#### Rotavirus vaccine

##### Monovalent Rotarix vaccine

Twenty-two studies reported using the monovalent (RV1) Rotarix vaccine to reduce the risk of rotavirus diseases. The monovalent rotavirus vaccine is given orally at 2 months and 4 months of age.

In Zimbabwe, Mujuru et al. [26] showed that the RV1 vaccine was protective against rotavirus of any severity by 61% and against severe rotavirus disease by 68%, in children younger than 5 years and at least 6 months. In Australia, Maguire et al. [27] showed that the two-dose RV1 vaccine was effective against 88.6%, 83.7%, and 78.7% in children aged 6 to 11 months, 1 to 3 years, and 4 to 9 years, respectively. The vaccine was effective against 89.5% of rotavirus disease in the first year which dropped to 77.05% at 5 to 10 years post vaccination [27]. In Kenya, a surveillance study by Wandera et al. [28] showed that children were protected by one and two doses of RV1 vaccines, with two doses being more effective than one dose. Hospitalization was reduced by 48% after the rotavirus vaccine was introduced in Kenya [28].

In Zambia, Mpabalwani et al. [29] showed that children younger than 5 years old had fewer hospitalizations due to rotavirus disease, with hospitalizations dropping by 40% in the first year and 29% in the fourth year after vaccination. In Bangladesh, a trial by Zaman et al. [30] showed that children younger than 2 years old who had the monovalent rotavirus vaccine had a lower incidence of rotavirus (29%), with higher effectiveness in the first year compared to the second year post vaccination. In villages that received the rotavirus vaccine, children had a lower incidence of Rotavirus disease, 2.8 per 100 person-years compared to 4.1 per 100 person-years in villages where the vaccine was not administered [30].

In Armenia, Sahakyan et al. [31] showed that when vaccinated, children between 0 and 59 months old had 48% fewer hospitalizations due to rotavirus in the first year after vaccination and ≥ 75% fewer hospitalizations in the 2nd and 3rd year post vaccination. Interestingly, unvaccinated children also had more than 30% fewer hospitalizations, suggesting other community-level factors affecting the incidence of rotavirus disease [31]. The two-dose monovalent vaccine reduced the incidence of rotavirus disease of any severity by 62% in children aged 6–23 months, 68% in those aged 6–11 months, and 60% in children aged 12–23 months [31]. In Moldova, the introduction of a vaccination program for children aged 6 months to 5 years, led to hospitalizations for rotavirus dropping from 45 to 25% and 14% in the first and second years, respectively [32]. The two-dose rotavirus vaccine was also effective in preventing 79% of rotavirus hospitalizations and 84% of hospitalization for severe disease [32]. The reduction in hospitalizations was also seen in unvaccinated children [32]. In Botswana, children older than 4 months who received the two-dose vaccine had 54% fewer hospitalizations after two doses and 48% fewer hospitalizations after one dose [33].

In Malawi, children younger than 5 years old, who received the monovalent rotavirus vaccine were 70.6% and 31.7% less likely to be hospitalized for rotavirus disease, in the first and second year of life, respectively, irrespective of nutritional status or HIV exposure [34]. In Malawi, the introduction of a rotavirus vaccination program for children younger than 5 years led to hospitalizations for rotavirus dropping from 50 to 40% and 31% in the years following vaccination introduction [35].

In Brazil, children between 4 and 24 months old, who received 2 doses of the RV1 vaccine had 72% fewer hospitalizations and those who received one dose had 62% fewer hospitalizations for rotavirus diarrhea [36]. In South Africa, children aged 18 to 23 months who received two doses of the RV1 vaccine had a 57% reduced risk of being hospitalized, while children who received one dose had a 40% reduced risk of being hospitalized for rotavirus diarrhea, irrespective of HIV exposure status [37].

In Bolivia, among children of at least 8 weeks old, those who had received the RV1 vaccine were 69% less likely to be hospitalized compared to rotavirus negative controls and 77% less likely to be hospitalized compared to non-diarrhea controls [38]. As with other studies, one dose of the RV1 vaccine resulted in protection, but to a lesser degree: 36% for negative controls and 56% for non-diarrhea controls [38]. In addition, the study showed sustained protection of the vaccination and protection of vaccine against various serotypes [38]. In Belgium, children of at least 14 weeks old, who received two doses of the RV1 vaccine had a 90% reduction in hospitalizations for rotavirus gastroenteritis [39]. The vaccine was also protective against 86% of co-infected cases (adenovirus, astrovirus, and/or norovirus) [39]. In Brazil, children who were at least 12 weeks old, who received the RV1 vaccination, had a 75.8% reduced risk of hospitalization compared to neighborhood controls and a 40% reduced risk compared to hospital controls [40].

In Zambia, children up to 5 years old, who were vaccinated with two doses of the RV1 vaccine, showed between 26 and 56% fewer hospitalizations depending on age [41]. In Tanzania, the introduction of a vaccination program led to reduced detection of rotavirus in children younger than 5 years old [42]. Children between 5 and 23 months old, who received one dose of the monovalent vaccine, showed 53% fewer hospitalizations, while those who received two doses of the vaccine showed 49% fewer hospitalizations [42]. In Canada, increased vaccine coverage in children between 8 weeks and 3 years old led to a 70.1% reduction in rotavirus prevalence, with a 1% increase in coverage leading to a 3.8% decrease in prevalence [43]. In Morocco, children younger than 5 years old who received the rotavirus vaccine were 41% less likely to be hospitalized [44].

In Colombia, children of at least 8 weeks old were vaccinated and followed up [45]. Between 6 and 11 months old, vaccine effectiveness was 79.19%, and 39.75% among children older than 1 year. Hospitalizations were reduced by 84.42% among children 6 to 11 months old, and by 79.49% among children older than 1 year [45]. In Nairobi, Kenya, the introduction of a vaccination program for children younger than 5 years led to a decline in rotavirus infections from 22.1% in 2015 to 14.8% in 2016 to 10% in 2017 [46]. In Italy, the introduction of a vaccination program led to a 49.2% reduction in hospitalizations for rotavirus disease [47].

##### Pentavalent Rotateq

Nine studies reported the use of pentavalent vaccine as an intervention for rotavirus. The vaccine is given to children at 2 months, 4 months, and 6 months.

In these children, vaccine effectiveness was 77% in children aged 6–59 months and 86% in children aged 6–23 months for children who received the full dose, while incomplete doses 72% and 75% protection for the respective age categories in a study done in Israel [48]. In Bukina Faso, the introduction of the vaccine resulted in reduced hospital admission from 36% in 2014 to 22% in 2015 to 20% in 2016 among children under the age of five [49]. The reduction in hospitalizations was even more pronounced for infants, dropping from 38% in 2014 to 21% in 2015 to 17% in 2016 [49]. In Burkina Faso, the full three-dose Rotateq (RV5) vaccine offered 58% protection against rotavirus hospitalization in children 6–11 months old and 19% in children older than 1 year [49].

In Finland, among children younger than 16 years old who received three doses of the RV5 vaccine, vaccine effectiveness was 92.1% [50]. In Finland, the introduction of the vaccination program led to a 78% reduction in hospitalizations [50]. In Israel, a surveillance study showed that vaccine effectiveness was 63% against emergency department (ED) visit or hospitalization for children between 6 months and 5 years old who had received the full vaccination schedule [51]. For different age groups, vaccine effectiveness was 64% for children aged 6–11 months and 71% for children between 12–23 months [49]. Vaccine effectiveness was 59% against hospitalization and 67% against ED visit [51].

In Nicaragua, vaccine effectiveness was reportedly 87% for children younger than 5 years old who had received three doses of the RV5 vaccine compared to community controls, 64% for hospital controls, and 76% when the groups were combined [52]. In France, the introduction of the RV5 vaccine led to the halving of hospitalizations within 2 years of vaccine introduction, and a risk reduction of 98% for hospitalizations for rotavirus diarrhea [53].

In Nicaragua, RV5 vaccination with 3 doses was associated with a lower risk of rotavirus diarrhea requiring overnight admission or intravenous hydration (odds ratio [OR] 0.54) and a progressively lower risk of severe (OR, 0.42) and very severe rotavirus diarrhea (OR, 0.23) [54]. In Nicaragua, the vaccine effectiveness of RV5 was 46% against rotavirus disease requiring admission or treatment with intravenous hydration, 58% against severe rotavirus diarrhea, and 77% against very severe rotavirus diarrhea [54]. In the USA, in children younger than 5 months old, vaccine effectiveness was 74% after one dose, 88% after two doses, and 87% after three doses [55]. For infants enrolled in the IVANHOE surveillance study, a RV5 vaccination program led to a 2.6- to 11-fold reduction in rotavirus hospitalizations for premature infants [56].

##### Rotarix and Rotateq

Sixteen studies reported on the combined use of the Rotarix and or Rotateq vaccine to mitigate the risk of Rotavirus at a community level.

In Japan, the RV1 and RV5 vaccines had a combined effectiveness of 70.4% against hospitalization due to rotavirus gastroenteritis in children younger than 5 years old [57]. In China, vaccine effectiveness for either rotavirus vaccine was 92% against hospitalization of children between 1 month and 5 years old [58]. In the USA, the RV1 and RV5 vaccines had similar effectiveness [59], with two doses of RV1 resulting in vaccine effectiveness of 84% among children aged 8–23 months and 82% among children older than 2 years old, against emergency department visits or inpatient care. For the same age groups, three RV5 doses had a vaccine effectiveness of 80% and 87%, respectively [59].

In Guatemala, the RV1 (63%) and RV5 (69%) vaccines were shown to have a similar effectiveness [60]. Combined vaccine effectiveness was 74% with hospital controls, and 52% with test-negative controls against visiting the emergency department or hospitalization [60]. In Portugal, vaccine efficacy was lower for at least one dose of RV1 (83.7%) compared to one dose of RV5 (96.1%) in a cohort of children between 8 weeks and 3 years old against acute gastroenteritis [61].

In Taiwan, two doses of the RV1 vaccine had an effectiveness of 90.4% and 92.5% with RV-negative acute gastroenteritis (AGE) and non-AGE controls, respectively, against hospitalization for rotavirus gastroenteritis for children between 8 months and 3 years old [62]. Three-dose RV5 had a greater effectiveness of 96.8% and 97.1% compared to RV-negative AGE and non-AGE controls, respectively [62]. In the USA, three doses of the RV5 vaccine (84%) than two doses of the RV1 vaccine (70%) prevented rotavirus-associated hospitalizations and emergency department visits of children younger than 5 years old [63]. In Spain, the RV1 and RV5 vaccines had similar effectiveness in preventing rotavirus gastroenteritis (78%) and hospitalization (83%) in children between 3 months and 5 years old [64].

In the USA, vaccine effectiveness against hospitalization with rotavirus gastroenteritis for at least one dose of vaccine was 94.3% for hospitalized controls and 96.9% for community controls [65]. In Saudi Arabia, the introduction of a national vaccination program reduced hospitalizations due to rotavirus-positive gastroenteritis from 38.5 to 13.2% and increased the median age of infection from 16 to 44 months [14].

In Japan, RV1 and RV5 vaccines had similar effectiveness of 80.6% and 80.4%, respectively [66]. Although vaccine effectiveness reduced with age, an effectiveness of greater than 70% was maintained up to 2 years after vaccination [66]. Vaccine effectiveness against severe gastroenteritis, requiring intravenous rehydration or hospitalization, was 97.3% [66]. In Lebanon, the combined vaccine effectiveness of the RV1 and RV5 vaccines was 68.4%, children who were rotavirus negative 21% more likely to be vaccinated compared to unvaccinated children who were rotavirus positive [67].

In the USA, combined vaccine effectiveness for full RV1 and RV5 vaccines was 80% in children younger than 8 years old [68]. In the USA, Mohammed et al. [69] showed that children were still protected if they received combined vaccines (single vaccine OR 0.21 vs combined OR 0.29). In Belgium, hospitalizations declined in children younger than 2 years old, in the first year after vaccination (65%) and the second year after vaccination (80%) [70]. For children younger than 2 months, hospitalizations declined by 50% and 64% in the first and second year post vaccination, respectively [70]. For children older than 2 years, hospitalizations declined by 20% in the first year post vaccination and by 64% in the second year post vaccination [70]. In the USA, a cohort study, linking stool samples with immunization records showed that vaccine effectiveness was similar for RV1 (91%) and RV5 (92%) [71].

##### Lanzhou lamb rotavirus vaccine

Two studies reported on the Lanzhou Lamb Rotavirus vaccine. Both were case-control studies involving children younger than 5 years, both conducted in China. The first study by Li et al. [72] found a vaccine effectiveness of one-dose vaccine versus zero vaccine to be 34.9%, 87.7% effective against severe disease, and 36.2% for children 2–35 months old. Fu et al. [73] found a vaccine effectiveness 44.3% for children 9–11 months old, 52.8% for children 12–17 months old, and 51.8% for children 18–35 months old for one dose [73].

#### Combined water supply, household water treatment, and safe storage

In Kinshasa, interventions to implement an emergency water supply, household water treatment and safe storage, home disinfection, and hygiene promotion led to a 71% reduction in cholera cases in 4 weeks among people 2 years and older [74].

#### Oral cholera vaccine

Four studies reported on two-dose oral cholera vaccines. In Guinea, two-dose vaccines were 86.6% effective in preventing cholera among cholera suspects older than 12 years [75]. In India, a two-dose and single-dose cholera vaccine was 69.0% and 33% effective, respectively [76]. In Haiti, the cumulative 4-year vaccine effectiveness of 2 doses was 76% and the predicted effectiveness of the single dose was 79%, which was not effective by the second year after vaccination [77]. In Tanzania, a two-dose vaccine for people older than 2 years old resulted in the protection of 79%; this protection seemed to extend to non-vaccinated individuals who stayed in households where neighbors had been vaccinated [78].

#### Water filtration

Only one study from Scotland reported on the use of enhanced filtration of drinking water as an intervention for cryptosporidiosis, where the incidence of cryptosporidiosis was associated with unfiltered water supply to homes (OR 1.86) [79].

#### Vi polysaccharide vaccination

At the community level, Vi polysaccharide vaccines have been tested against typhoid in one study. In India, the incidence of *Salmonella typhi* (*S. typhi*) and *Salmonella paratyphi* (*S. paratyphi*) reduced after 2 years of vaccination from 194/100,000 and 104/100,000 to 190/100,000 and 170/100,000, respectively [80].

## Discussion

In this review, we retrieved 56 studies, mostly case-control studies that reported on interventions to reduce the incidence or prevalence of waterborne diarrheal diseases at a community level. Waterborne diarrheal diseases included rotavirus, cholera, typhoid, and cryptosporidiosis with most of the studies reporting rotavirus diseases. Interventions included rotavirus vaccines (monovalent, pentavalent, and Lanzhou Lamb), emergency water supply, household water treatment and safe storage, home disinfection, and hygiene promotion. Other studies reported on two-dose cholera vaccines, enhanced water filtration for cryptosporidiosis and Vi polysaccharide vaccine for typhoid. The identified studies mostly reported interventions targeting children younger than 5 years old. Most of the studies were conducted in the USA, in high-income countries, and developing countries. In addition, some studies reported indirect effects of the interventions on reducing the risk of diseases. Across the world, diarrheal diseases are commonly caused by waterborne pathogens. Diarrheal disease is a leading cause of mortality among children in developing countries [81–83]. Young children generally have poor immunity to diarrheal disease due to their poorly developed digestive system and higher risk of dehydration. In developing countries, exposure to unsafe drinking water, poor sanitation, and hygiene may also contribute to increased risk [84]. In the USA, rotavirus was the major cause of severe diarrhea among children before vaccines were developed [85]. The development of vaccines may explain why so many studies have been conducted in the USA. Rotavirus is common in developing countries, including African countries, and vaccines have led to a reduced burden of disease in these areas [86, 87]. The disproportionate risk of children to diarrheal disease may also explain why most studies focus on this age group.

Most of the interventions, identified at the community level, focused on the effectiveness of rotavirus vaccines. The review identified studies that reported three (3) types of vaccine for rotavirus including Rotarix (RV1), Rotateq (RV5), and Lanzhou Lamb. These are the three most commonly available rotavirus vaccines [82, 88]. These vaccines have been successfully implemented across the world (Supplementary Table 3) and have reduced hospitalizations of infants requiring rehydration in many countries [89, 90]. Our review revealed that cholera is a common problem in lower middle-income and low-income countries or developing countries such as the Democratic Republic of Congo, Tanzania, Guinea, Haiti, and India [91]. In these countries, the high incidence of cholera can be attributed to poor provision of water, sanitation and hygiene facilities, and poor health care systems [92, 93]. We identified studies that tested the effectiveness of a two-dose cholera vaccine, which was effective in people older than 12. One dose was less effective than two doses in protecting against cholera infection [94]. Governments of developing countries must aim to improve water supply, sanitation, and hygiene facilities to control cholera outbreaks.

The limited time frame of our search may explain why vaccines featured so heavily in the returned results. One study, from Kinshasa, DRC, investigated the use of combined interventions including emergency water supply, household water treatment with chlorine and safe storage, home disinfection, and hygiene promotion activities and accessories such as soap and health messages to households to reduce cholera cases. Taylor et al. [95] also reported that a combination of interventions, including treat at point of use, hygiene promotion, water storage in disinfected vessels, and household disinfections helped to control cholera outbreaks in developing countries. Improving sanitation infrastructure such as toilets can also help to control cholera outbreaks, especially in developing countries that lack basic infrastructure and do not always have access to vaccines.

Recently, one study from Scotland reported using advanced water filtration techniques to remove cryptosporidiosis from the household water supply. In the USA, Betancourt and Rose [96] also reported using ultra- and microfiltration to remove *Cryptosporidium* cysts from the water supply. These tiny cysts cannot be removed from water using standard water treatment techniques [97]. Identifying and treating water-borne pathogens at a community level is important, because different pathogens will require different interventions.

We reviewed several studies that showed indirect effects (herd immunity) of various interventions to reduce the risks of waterborne diarrheal diseases. In Moldova and Bolivia, Gheorghita et al. [32] and Patel et al. [38] reported a drop in hospitalizations among vaccinated and unvaccinated children. This has been noticed before where both RV1 and RV2 had indirect effects on unvaccinated people [98]. Similar effects were reported for cholera vaccines and the Lanzhou Lamb vaccines [99].

### Limitations

We did not review articles written in other languages. Thus, we may have missed important studies from non-English speaking populations, a possible cause of bias. We did, however, screen the titles and abstracts of the articles that were found in other languages during the search but none of the articles met the inclusion criteria. We also searched different databases to ensure that a variety of studies were included in the review. We however did not search important databases like EMBASE and Global Health due to a lack of access to the stated databases. However, our searches were not restricted to a particular region or area; as such, this helped to widen the coverage. We did not conduct a meta-analysis; however, the qualitative narratives give a general idea of the existing interventions to mitigate the risks of waterborne diarrhea diseases at a community level.

We only included studies that reported interventions with positive outcomes. This was important because we wanted to identify effective interventions for mitigating the risk of waterborne diseases.

The inclusion of different types of study designs most of which cannot conclude causation is a limitation. Further, the reviewed studies included were all peer reviewed to ensure quality.

## Conclusions

Currently, several interventions exist to mitigate the risk of waterborne diarrhea diseases including vaccines for rotavirus diseases (monovalent, pentavalent, and Lanzhou lamb Vaccines), 2-dose cholera vaccines, water supply, household water treatment and safe storage, home disinfection, and hygiene promotion for cholera, enhanced filtration of water for cryptosporidiosis, and use of Vi polysaccharide vaccine for typhoid. Our results show that interventions for waterborne diseases must be concentrated in developing countries as they are the main areas where these diseases are most common. The interventions must also concentrate mostly on control of the disease in children even though adults are also affected. At a community level, vaccines seem to be the most effective interventions and are probably the easiest to implement.

## Supplementary Information

**Additional file 1: Supplementary Table 1.** Search Strategy for review of interventions to mitigate risk of waterborne diarrheal diseases.

**Additional file 2: Appendix 1.** Included and Excluded Studies with Reasons.

**Additional file 3: Supplementary Table 2.** Characteristics of studies included in the systematic review.

**Additional file 4: Supplementary Table 3.** Objectives, participants, interventions and results of the studies included in the systematic review.

## Data Availability

All the studies included in this review are available online.

## Abbreviations

JBI: Joanna Briggs Institute (JBI)
PRISMA: Preferred Reporting Items for Systematic Reviews and Meta-Analyses
CI: Confidence interval
SDG: Sustainable Development Goal
WHO: World Health Organization
ETEC: Enterotoxigenic *Escherichia coli*
RV1: Monovalent or Rotarix rotavirus vaccine
RV5: Pentavalent or RotaTeq rotavirus vaccine
VE: Vaccine effectiveness
OR: Odds ratio
