# Supplementary material for: Community-level interventions for mitigating the risk of waterborne diarrheal diseases: a systematic review
Source: Syst Rev. 2022 Apr 18;11:73. doi: 10.1186/s13643-022-01947-y (PMC9016942; doi:10.1186/s13643-022-01947-y)
Supplement: Supplementary file 1 — Additional file 1: Supplementary Table 1. Search Strategy for review of interventions to mitigate risk of waterborne diarrheal diseases. [file 13643_2022_1947_MOESM1_ESM.docx]

**Supplementary Table 1. Search Strategy for review of interventions to mitigate risk of waterborne diarrheal diseases**

| **Database and Date Last Searched** | **Search Terms/Phrases** | **Limiters** | **Results** |
| --- | --- | --- | --- |
| **CINAHL** |  |  |  |
| 02/08/2020 | “waterborne disease” OR “waterborne infection” OR “waterborne illness” OR “waterborne outbreak” OR" waterborne sickness” (Free search) | Published Date: 20090101-20201231 | 91 |
| 02/08/2020 | (MH "Cholera/PC") MH (Major Headings)  PC (prevention/control) | Published Date: 20090101-20201231 | 266 |
| 02/08/2020 | (MH "Rotavirus Infections/PC") | Published Date: 20090101-20201231 | 520 |
| 02/08/2020 | Reovirus* (Free search) | Published Date: 20090101-20201231 | 53 |
| 02/08/2020 | (MH "Escherichia Coli Infections/PC") | Published Date: 20090101-20201231 | 176 |
| 02/08/2020 | (MH "Enterovirus Infections/PC") | Published Date:  20090101-20201231 | 46 |
| 02/08/2020 | (MH "Caliciviridae Infections/PC") | Published Date: 20090101-20201231 | 172 |
| 02/08/2020 | Astrovirus* (Free search) | Published Date: 20090101-20201231 | 66 |
| 02/08/2020 | (MH "Adenoviruses") | Published Date: 20090101-20201231 | 23 |
| 02/08/2020 | (MH "Giardiasis/PC") | Published Date: 20090101-20201231 | 16 |
| 02/08/2020 | Cyclosporia* Free search | Published Date: 20090101-20201231 | 31 |
| 02/08/2020 | (MH “Dysentery, Bacillary/PC”) | Published Date: 20090101-20201231 | 31 |
| 02/08/2020 | (MH “Dysentery/PC”) | Published Date: 20090101-20201231 | 12 |
| 02/08/2020 | (MH "Cryptosporidiosis/PC") | Published Date: 20090101-20201231 | 27 |
| 02/08/2020 | (MH "Yersinia Infections/PC") | Published Date: 20090101-20201231 | 3 started 2011 |
| 02/08/2020 | (MH "Shigella") | Published Date: 20090101-20201231 | 316 |
| 02/08/2020 | (MH "Typhoid/PC") | Published Date: 20090101-20201231 | 201 |
| 02/08/2020 | (MH "Campylobacter Infections/PC") | Published Date: 20090101-20201231 | 33 |
| 02/08/2020 | (MH "Amebiasis/PC") | Published Date: 20090101-20201231 | 13 |
| **Total** |  |  | **2096** |
| **Scopus** |  |  |  |
| 03/08/2020 | TITLE-ABS-KEY (waterborne) SEARCH #1 | NONE | 15,418 |
| 03/08/2020 | disease* OR infect* OR illness* OR outbreak* OR sickness*  TITLE-ABS-KEY (disease* OR infect* OR illness* OR outbreak* OR sickness* )  #2 | NONE | 11,078,068 |
| 03/08/2020 | #1 AND #2  (TITLE-ABS-KEY ( waterborne ) ) AND ( TITLE-ABS-KEY ( disease* OR infect* OR illness* OR outbreak* OR sickness* ) ) | Published date: 2009 -2020 | 3,241 |
| 03/08/2020 | TITLE-ABS-KEY (cholera OR rotavirus* OR reovirus* OR shigell* OR enterovirus* OR calicivir* OR norovirus* OR astrovirus* OR adenovirus* OR giard* OR cyclosporia* OR dysenter* OR cryptosporid* OR yersin* OR salmonell* OR typhoid* OR campylobacter* OR amoebia* )  #1 | NONE | 391,463 |
| 03/08/2020 | Intervention*(TITLE-ABS-KEY)  #2 | NONE | 1,449,978 |
| 03/08/2020 | (TITLE-ABS-KEY (cholera OR rotavirus* OR reovirus* OR shigell* OR enterovirus* OR calicivir* OR norovirus* OR astrovirus* OR adenovirus* OR giard* OR cyclosporia* OR dysenter* OR cryptosporid* OR yersin* OR salmonell* OR typhoid* OR campylobacter* OR amoebia* ) ) AND ( TITLE-ABS-KEY ( intervention* ) )  #1 AND #2 | Published Date: 2009 to 2020 | 3,993 |
| **Total** |  |  | **7234** |
| **Pubmed** |  |  |  |
| 02/08/2020 | Waterborne [Title/Abstract] #1 | 2009-2020 | 7,292 |
| 02/08/2020 | "disease*"[Title/Abstract] OR "infect*"[Title/Abstract] OR "illness*"[Title/Abstract] OR "sickness*"[Title/Abstract] OR "outbreak*"[Title/Abstract] #2 | 2009 to 2020 | 5,398,395 |
| 02/08/2020 | "Waterborne"[Title/Abstract] AND (((("disease*"[Title/Abstract] OR "infect*"[Title/Abstract]) OR "illness*"[Title/Abstract]) OR "sickness*"[Title/Abstract]) OR "outbreak*"[Title/Abstract]) #1 AND #2 | 2009-2020 | 2,285 |
| 02/08/2020 | “waterborne diseases/prevention and control”[MeSH Terms] | 2009 to 2020 | 43 started in 2014 |
| 02/08/2020 | “Cholera/prevention and control”[Mesh] | 2009 TO 2020 | 637 |
| 02/08/2020 | “Rotavirus infections/prevention and control”[Mesh] | 2009-2020 | 1,363 |
| 02/08/2020 | “ Reoviridae Infections/prevention and control”[Mesh] | 2009-2020 | 1,650 |
| 02/08/2020 | “Escherichia coli Infections/prevention and control”[Mesh] |  | 917 |
| 02/08/2020 | “Enterovirus infections/prevention and control”[Mesh] | 2009-2020 | 2,359 |
| 02/08/2020 | “Caliciviridae Infections/prevention and control”[Mesh] | 2009-2020 | 323 |
| 02/08/2020 | “Mamastrovirus”[Mesh] | 2009 -2020 | 259 |
| 02/08/2020 | “Adenoviridae Infections/prevention and control”[Mesh] | 2009-2020 | 124 |
| 02/08/2020 | “Giardiasis/prevention and control”[Mesh] | 2009-2020 | 61 |
| 02/08/2020 | “Cyclosporiasis/prevention and control”[Mesh] | 2009-2020 | 2 |
| 02/08/2020 | “Dysentery/prevention and control”[Mesh] | 2009-2020 | 196 |
| 31/07/2020  02/08/2020 | “Dysentery, Amebic/prevention and control”[Mesh] | 2009-2020 | 9 |
| 31/07/2020 | “Dysentery, Bacillary/prevention and control”[Mesh] | 2009-2020 | 144 |
| 31/07/2020 | “Cryptosporidiosis/prevention and control”[Mesh] | 2009-2020 | 133 |
| 31/07/2020 | “Yersinia Infections/prevention and control”[Mesh] | 2009-2020 | 319 |
| 31/07/2020 | “Salmonella infections/prevention and control”[Mesh] | 2009-2020 | 1,352 |
| 31/07/2020 | “Typhoid fever/prevention and control”[Mesh] | 2009-2020 | 314 |
| 31/07/2020 | “Campylobacter Infections/prevention and control”[Mesh] | 2009-2020 | 185 |
| **Total** |  |  | **12675** |
| **Web of science core collection** |  |  |  |
| 03/08/2020 | Waterborne #1 | NONE | 12,793 |
| 03/08/2020 | disease* OR infect* OR illness*  OR outbreak* OR sickness* #2 | NONE | 5,874,468 |
| 03/08/2020 | #1 AND #2 | 2009-2020 | 2815 |
| 03/08/2020 | TOPIC: (cholera OR rotavirus* OR reovirus* OR shigell* OR enterovirus* OR calicivir* OR norovirus* OR astrovirus* OR adenovirus* OR giard* OR cyclosporia* OR dysenter* OR cryptosporid* OR yersin* OR salmonell* OR typhoid* OR campylobacter* OR amoebia*) #1 | NONE | 289,176 |
| 03/08/2020 | TOPIC: (intervention*) #2 | NONE | 1,175,146 |
| 03/08/2020 | #1 AND #2 | 2009-2020 | 3,433 |
| **Total** |  |  | **6248** |
| **Cochrane library** |  |  |  |
| 01/08/2020 | Waterborne AND (disease* OR infect* OR illness* sickness* OR outbreak*) Title and Abstract[tiab] | 2009-2020 | 27 |
| 01/08/2020 | “Waterborne Diseases/prevention and control”[Mesh] | 2014 to 2020 | 0 |
| 01/08/2020 | “Cholera/prevention and control”[Mesh] |  | 47 |
| 01/08/2020 | “Rotavirus infections/prevention and control”[Mesh] | 2009-2020 | 96 |
| 01/08/2020 | “ Reoviridae Infections/prevention and control”[Mesh] | 2009-2020 | 96 |
| 01/08/2020 | “Escherichia coli Infections/prevention and control”[Mesh] | 2009-2020 | 26 |
| 01/08/2020 | “Enterovirus infections/prevention and control”[Mesh] | 2009-2020 | 150 |
| 01/08/2020 | “Caliciviridae Infections/prevention and control”[Mesh] | 2009-2020 | 13 |
| 01/08/2020 | “Mamastrovirus”[Mesh] | 2009-2020 |  |
| 01/08/2020 | “Adenoviridae Infections/prevention and control”[Mesh] | 2009-2020 | 3 |
| 01/08/2020 | “Giardiasis/prevention and control”[Mesh] | 2009 TO 2020 | 3 |
| 01/08/2020 | “Cyclosporiasis/prevention and control”[Mesh] | 2009 TO 2020 | 0 |
| 01/08/2020 | “Dysentery/prevention and control”[Mesh] |  | 9 |
| 01/08/2020 | “Cryptosporidiosis/prevention and control”[Mesh] | 2009-2020 | 2 |
| 01/08/2020 | “Yersinia Infections/prevention and control”[Mesh] | 2009-2020 | 2 |
| 01/08/2020 | “Salmonella infections/prevention and control”[Mesh] | 2009-2020 | 24 |
| 01/08/2020 | “Campylobacter Infections/prevention and control”[Mesh] | 2009-2020 | 22 |
| **Total** |  |  | **520** |
| **GRAND TOTAL** |  |  | **28773** |
