# Supplementary material for: Community-level interventions for mitigating the risk of waterborne diarrheal diseases: a systematic review
Source: Syst Rev. 2022 Apr 18;11:73. doi: 10.1186/s13643-022-01947-y (PMC9016942; doi:10.1186/s13643-022-01947-y)
Supplement: Supplementary file 2 — Additional file 2: Appendix 1. Included and Excluded Studies with Reasons. [file 13643_2022_1947_MOESM2_ESM.docx]

**Appendix 1: Included and Excluded Studies with Reasons**

**Included Studies**

1. Bompangue D, Moore S, Taty N, Impouma B, Sudre B, Manda R, et al. Description of the targeted water supply and hygiene response strategy implemented during the cholera outbreak of 2017-2018 in Kinshasa, DRC. BMC Infect Dis. 2020;20(1).

2. Mujuru HA, Burnett E, Nathoo KJ, Ticklay I, Gonah NA, Mukaratirwa A, et al. Monovalent Rotavirus Vaccine Effectiveness Against Rotavirus Hospitalizations Among Children in Zimbabwe. Clin Infect Dis. 2019;69(8):1339-44.

3. Maguire JE, Glasgow K, Glass K, Roczo-Farkas S, Bines JE, Sheppeard V, et al. Rotavirus Epidemiology and Monovalent Rotavirus Vaccine Effectiveness in Australia: 2010-2017. Pediatrics. 2019;144(4).

4. Li J, Zhang Y, Yang Y, Liang Z, Tian Y, Liu B, et al. Effectiveness of Lanzhou lamb rotavirus vaccine in preventing gastroenteritis among children younger than 5 years of age. Vaccine. 2019;37(27):3611-6.

5. Gikonyo J, Mbatia B, Okanya P, Obiero G, Sang C, Nyangao J. Rotavirus prevalence and seasonal distribution post vaccine introduction in Nairobi county Kenya. Pan Afr Med J. 2019;33:269.

6. Wandera EA, Mohammad S, Bundi M, Nyangao J, Galata A, Kathiiko C, et al. Impact of rotavirus vaccination on rotavirus hospitalisation rates among a resource-limited rural population in Mbita, Western Kenya. Trop Med Int Health. 2018;23(4):425-32.

7. Restivo V, Caracci F, Sannasardo CE, Scarpitta F, Vella C, Ventura G, et al. Rotavirus gastroenteritis hospitalization rates and correlation with rotavirus vaccination coverage in Sicily. Acta Biomed. 2018;89(3):437-42.

8. Muhsen K, Anis E, Rubinstein U, Kassem E, Goren S, Shulman LM, et al. Effectiveness of rotavirus pentavalent vaccine under a universal immunization programme in Israel, 2011-2015: a case-control study. Clin Microbiol Infect. 2018;24(1):53-9.

9. Mpabalwani EM, Simwaka JC, Mwenda JM, Matapo B, Parashar UD, Tate JE. Sustained impact of rotavirus vaccine on rotavirus hospitalisations in Lusaka, Zambia, 2009-2016. Vaccine. 2018;36(47):7165-9.

10. Jani B, Hokororo A, McHomvu J, Cortese MM, Kamugisha C, Mujuni D, et al. Detection of rotavirus before and after monovalent rotavirus vaccine introduction and vaccine effectiveness among children in mainland Tanzania. Vaccine. 2018;36(47):7149-56.

11. Franke MF, Ternier R, Jerome JG, Matias WR, Harris JB, Ivers LC. Long-term effectiveness of one and two doses of a killed, bivalent, whole-cell oral cholera vaccine in Haiti: an extended case-control study. The Lancet Global health. 2018;6(9):e1028-e35.

12. Bonkoungou IJO, Aliabadi N, Leshem E, Kam M, Nezien D, Drabo MK, et al. Impact and effectiveness of pentavalent rotavirus vaccine in children <5 years of age in Burkina Faso. Vaccine. 2018;36(47):7170-8.

13. Araki K, Hara M, Tsugawa T, Shimanoe C, Nishida Y, Matsuo M, et al. Effectiveness of monovalent and pentavalent rotavirus vaccines in Japanese children. Vaccine. 2018;36(34):5187-93.

14. Zaman K, Sack DA, Neuzil KM, Yunus M, Moulton LH, Sugimoto JD, et al. Effectiveness of a live oral human rotavirus vaccine after programmatic introduction in Bangladesh: a cluster-randomized trial. PLoS Med. 2017;14(4):e1002282.

15. Yoshiyuki F, Atsuko N, Shinobu M, Haruka I, Toyoko N, Osamu N, et al. Effectiveness of rotavirus vaccines against hospitalisations in Japan. BMC Pediatr. 2017;17:1-7.

16. Yeung KHT, Tate JE, Chan CC, Chan MCW, Chan PKS, Poon KH, et al. Rotavirus vaccine effectiveness in Hong Kong children. Vaccine. 2016;34(41):4935-4

17. Sahakyan G, Grigoryan S, Wasley A, Mosina L, Sargsyan S, Asoyan A, et al. Impact and Effectiveness of Monovalent Rotavirus Vaccine in Armenian Children. Clin Infect Dis. 2016;62 Suppl 2:S147-54.

18. Leshem E, Givon-Lavi N, Tate JE, Greenberg D, Parashar UD, Dagan R. Real-World Effectiveness of Pentavalent Rotavirus Vaccine Among Bedouin and Jewish Children in Southern Israel. Clin Infect Dis. 2016;62 Suppl 2:S155-60.

19. Immergluck LC, Parker TC, Jain S, Laghaie E, Spandorfer P, Jerris RC, et al. Sustained Effectiveness of Monovalent and Pentavalent Rotavirus Vaccines in Children. J Pediatr. 2016;172:116-20.e1.

20. Gheorghita S, Birca L, Donos A, Wasley A, Birca I, Cojocaru R, et al. Impact of Rotavirus Vaccine Introduction and Vaccine Effectiveness in the Republic of Moldova. Clin Infect Dis. 2016;62 Suppl 2:S140-6.

21. Gastañaduy PA, Steenhoff AP, Mokomane M, Esona MD, Bowen MD, Jibril H, et al. Effectiveness of Monovalent Rotavirus Vaccine After Programmatic Implementation in Botswana: A Multisite Prospective Case-Control Study. Clin Infect Dis. 2016;62 Suppl 2:S161-7.

22. Gastañaduy PA, Contreras-Roldán I, Bernart C, López B, Benoit SR, Xuya M, et al. Effectiveness of Monovalent and Pentavalent Rotavirus Vaccines in Guatemala. Clin Infect Dis. 2016;62 Suppl 2:S121-6.

23. Beres LK, Tate JE, Njobvu L, Chibwe B, Rudd C, Guffey MB, et al. A Preliminary Assessment of Rotavirus Vaccine Effectiveness in Zambia. Clin Infect Dis. 2016;62 Suppl 2:S175-82.

24. Bar-Zeev N, Jere KC, Bennett A, Pollock L, Tate JE, Nakagomi O, et al. Population Impact and Effectiveness of Monovalent Rotavirus Vaccination in Urban Malawian Children 3 Years After Vaccine Introduction: Ecological and Case-Control Analyses. Clin Infect Dis. 2016;62 Suppl 2(Suppl 2):S213-9.

25. Ali Z, Harastani H, Hammadi M, Reslan L, Ghanem S, Hajar F, et al. Rotavirus Genotypes and Vaccine Effectiveness from a Sentinel, Hospital-Based, Surveillance Study for Three Consecutive Rotavirus Seasons in Lebanon. PLoS One. 2016;11(8):e0161345.

26. Wierzba TF, Kar SK, Mogasale VV, Kerketta AS, You YA, Baral P, et al. Effectiveness of an oral cholera vaccine campaign to prevent clinically-significant cholera in Odisha State, India. Vaccine. 2015;33(21):2463-9.

27. Payne DC, Selvarangan R, Azimi PH, Boom JA, Englund JA, Staat MA, et al. Long-term Consistency in Rotavirus Vaccine Protection: RV5 and RV1 Vaccine Effectiveness in US Children, 2012-2013. Clin Infect Dis. 2015;61(12):1792-9.

28. Mohammed A, Immergluck L, Parker TC, Jain S, Leong T, Anderson EJ, et al. Association between mixed rotavirus vaccination types of infants and rotavirus acute gastroenteritis. Vaccine. 2015;33(42):5670-7.

29. Marlow R, Ferreira M, Cordeiro E, Trotter C, Januário L, Finn A, et al. Case control study of rotavirus vaccine effectiveness in Portugal during 6 years of private market use. Pediatr Infect Dis J. 2015;34(5):509-12.

30. Doll MK, Buckeridge DL, Morrison KT, Gagneur A, Tapiero B, Charest H, et al. Effectiveness of monovalent rotavirus vaccine in a high-income, predominant-use setting. Vaccine. 2015;33(51):7307-14.

31. Benhafid M, Elomari N, Azzouzi Idrissi M, Rguig A, Gentsch JR, Parashar U, et al. Effect of monovalent rotavirus vaccine on rotavirus disease burden and circulating rotavirus strains among children in Morocco. J Med Virol. 2015;87(6):944-53.

32. Bar-Zeev N, Kapanda L, Tate JE, Jere KC, Iturriza-Gomara M, Nakagomi O, et al. Effectiveness of a monovalent rotavirus vaccine in infants in Malawi after programmatic roll-out: an observational and case-control study. Lancet Infect Dis. 2015;15(4):422-8.

33. Roué JM, Nowak E, Le Gal G, Lemaitre T, Oger E, Poulhazan E, et al. Impact of rotavirus vaccine on premature infants. Clin Vaccine Immunol. 2014;21(10):1404-9.

34. Pollock KG, Young D, Robertson C, Ahmed S, Ramsay CN. Reduction in cryptosporidiosis associated with introduction of enhanced filtration of drinking water at Loch Katrine, Scotland. Epidemiol Infect. 2014;142(1):56-62.

35. Cotes-Cantillo K, Paternina-Caicedo A, Coronell-Rodríguez W, Alvis-Guzmán N, Parashar UD, Patel M, et al. Effectiveness of the monovalent rotavirus vaccine in Colombia: a case-control study. Vaccine. 2014;32(25):3035-40.

36. Chang WC, Yen C, Wu FT, Huang YC, Lin JS, Huang FC, et al. Effectiveness of 2 rotavirus vaccines against rotavirus disease in Taiwanese infants. Pediatr Infect Dis J. 2014;33(3):e81-6.se

37. Vesikari T, Uhari M, Renko M, Hemming M, Salminen M, Torcel-Pagnon L, et al. Impact and effectiveness of RotaTeq® vaccine based on 3 years of surveillance following introduction of a rotavirus immunization program in Finland. Pediatr Infect Dis J. 2013;32(12):1365-73.

38. Ichihara MY, Rodrigues LC, Teles Santos CA, Teixeira Mda G, De Jesus SR, Alvim De Matos SM, et al. Effectiveness of rotavirus vaccine against hospitalized rotavirus diarrhea: A case-control study. Vaccine. 2014;32(23):2740-7.

39. Groome MJ, Page N, Cortese MM, Moyes J, Zar HJ, Kapongo CN, et al. Effectiveness of monovalent human rotavirus vaccine against admission to hospital for acute rotavirus diarrhoea in South African children: a case-control study. Lancet Infect Dis. 2014;14(11):1096-104.

40. Payne DC, Boom JA, Staat MA, Edwards KM, Szilagyi PG, Klein EJ, et al. Effectiveness of pentavalent and monovalent rotavirus vaccines in concurrent use among US children <5 years of age, 2009-2011. Clin Infect Dis. 2013;57(1):13-20.

41. Patel MM, Patzi M, Pastor D, Nina A, Roca Y, Alvarez L, et al. Effectiveness of monovalent rotavirus vaccine in Bolivia: case-control study. BMJ. 2013;346:f3726.

42. Cortese MM, Immergluck LC, Held M, Jain S, Chan T, Grizas AP, et al. Effectiveness of monovalent and pentavalent rotavirus vaccine. Pediatrics. 2013;132(1):e25-33.

43. Khatib AM, Ali M, von Seidlein L, Kim DR, Hashim R, Reyburn R, et al. Effectiveness of an oral cholera vaccine in Zanzibar: findings from a mass vaccination campaign and observational cohort study. Lancet Infect Dis. 2012;12(11):837-44.

44. Castilla J, Beristain X, Martínez-Artola V, Navascués A, García Cenoz M, Alvarez N, et al. Effectiveness of rotavirus vaccines in preventing cases and hospitalizations due to rotavirus gastroenteritis in Navarre, Spain. Vaccine. 2012;30(3):539-43.

45. Braeckman T, Van Herck K, Meyer N, Pirçon J-Y, Soriano-Gabarró M, Heylen E, et al. Effectiveness of rotavirus vaccination in prevention of hospital admissions for rotavirus gastroenteritis among young children in Belgium: case-control study. BMJ: British Medical Journal (Clinical Research Edition). 2012;345:e4752-e.

46. Raes M, Strens D, Vergison A, Verghote M, Standaert B. Reduction in pediatric rotavirus-related hospitalizations after universal rotavirus vaccination in Belgium. Pediatr Infect Dis J. 2011;30(7):e120-5.

47. Mast TC, Khawaja S, Espinoza F, Paniagua M, Del Carmen LP, Cardellino A, et al. Case-control study of the effectiveness of vaccination with pentavalent rotavirus vaccine in Nicaragua. Pediatr Infect Dis J. 2011;30(11):e209-15.

48. Justino MC, Linhares AC, Lanzieri TM, Miranda Y, Mascarenhas JD, Abreu E, et al. Effectiveness of the monovalent G1P[8] human rotavirus vaccine against hospitalization for severe G2P[4] rotavirus gastroenteritis in Belém, Brazil. Pediatr Infect Dis J. 2011;30(5):396-401.

49. Gagneur A, Nowak E, Lemaitre T, Segura JF, Delaperrière N, Abalea L, et al. Impact of rotavirus vaccination on hospitalizations for rotavirus diarrhea: the IVANHOE study. Vaccine. 2011;29(21):3753-9.

50. Ali M, Sur D, Kim DR, Kanungo S, Bhattacharya SK, Manna B, et al. Impact of Vi vaccination on spatial patterns of typhoid fever in the slums of Kolkata, India. Vaccine. 2011;29(48):9051-6.

51. Desai SN, Esposito DB, Shapiro ED, Dennehy PH, Vázquez M. Effectiveness of rotavirus vaccine in preventing hospitalization due to rotavirus gastroenteritis in young children in Connecticut, USA. Vaccine. 2010;28(47):7501-6.

52. Patel M, Pedreira C, De Oliveira LH, Tate J, Orozco M, Mercado J, et al. Association between pentavalent rotavirus vaccine and severe rotavirus diarrhea among children in Nicaragua. JAMA: Journal of the American Medical Association. 2009;301(21):2243-51.

53. Zaki A, Abousekkien M, Alkholy UM, Eid A. Effectiveness and impact of rotavirus vaccines in Saudi Arabia: A single hospital-based study. Arab J Gastroenterol. 2017;18(3):140-3.

54. Fu C, He Q, Xu J, Xie H, Ding P, Hu W, et al. Effectiveness of the Lanzhou lamb rotavirus vaccine against gastroenteritis among children. Vaccine. 2012;31(1):154-8.

55. Luquero FJ, Grout L, Ciglenecki I, Sakoba K, Traore B, Heile M, et al. Use of Vibrio cholerae vaccine in an outbreak in Guinea. N Engl J Med. 2014;370(22):2111-20.

56. Staat MA, Payne DC, Donauer S, Weinberg GA, Edwards KM, Szilagyi PG, et al. Effectiveness of pentavalent rotavirus vaccine against severe disease. Pediatrics 2011;128:e267-75.

**Excluded studies with reasons**

**Study not at community Level (ie school, health facility, household or school )**

1. Aliabadi N, Antoni S, Mwenda JM, Weldegebriel G, Biey JNM, Cheikh D, et al. Global impact of rotavirus vaccine introduction on rotavirus hospitalisations among children under 5 years of age, 2008-16: findings from the Global Rotavirus Surveillance Network. The Lancet Global health. 2019;7(7):e893-e903.

2. Weldegebriel G, Mwenda JM, Chakauya J, Daniel F, Masresha B, Parashar UD, et al. Impact of rotavirus vaccine on rotavirus diarrhoea in countries of East and Southern Africa. Vaccine. 2018;36(47):7124-30.

3. Troeger C, Khalil IA, Rao PC, Cao S, Blacker BF, Ahmed T, et al. Rotavirus Vaccination and the Global Burden of Rotavirus Diarrhea Among Children Younger Than 5 Years. JAMA Pediatrics. 2018;172(10):958-65.

4. Mwenda JM, Parashar UD, Cohen AL, Tate JE. Impact of rotavirus vaccines in Sub-Saharan African countries. Vaccine. 2018;36(47):7119-23.

5. M'Bangombe M, Pezzoli L, Reeder B, Kabuluzi S, Msyamboza K, Masuku H, et al. Oral cholera vaccine in cholera prevention and control, Malawi. Bull World Health Organ. 2018;96(6):428-35.

6. Santos PRD, Daniel LA. Occurrence and removal of Giardia spp. cysts and Cryptosporidium spp. oocysts from a municipal wastewater treatment plant in Brazil. Environmental Technology (United Kingdom). 2017;38(10):1245-54.

7. Markkula J, Hemming-Harlo M, Salminen MT, Savolainen-Kopra C, Pirhonen J, Al-Hello H, et al. Rotavirus epidemiology 5-6 years after universal rotavirus vaccination: persistent rotavirus activity in older children and elderly. Infect Dis (Lond). 2017;49(5):388-95.

8. Uhlig U, Kostev K, Schuster V, Koletzko S, Uhlig HH. Impact of rotavirus vaccination in Germany: rotavirus surveillance, hospitalization, side effects and comparison of vaccines. Pediatr Infect Dis J. 2014;33(11):e299-304.

9. Mahamud A, Kamadjeu R, Webeck J, Mbaeyi C, Baranyikwa MT, Birungi J, et al. Effectiveness of oral polio vaccination against paralytic poliomyelitis: a matched case-control study in Somalia. J Infect Dis. 2014;210 Suppl 1:S187-93.

10. Tate JE, Haynes A, Payne DC, Cortese MM, Lopman BA, Patel MM, et al. Trends in national rotavirus activity before and after introduction of rotavirus vaccine into the national immunization program in the United States, 2000 to 2012. Pediatr Infect Dis J. 2013;32(7):741-4.

11. Amburgey JE. Removal of cryptosporidium-sized polystyrene microspheres from swimming pool water with a sand filter with and without added perlite filter media. Journal of Environmental Engineering. 2011;137(12):1205-8.

12. Zeller M, Rahman M, Heylen E, De Coster S, De Vos S, Arijs I, et al. Rotavirus incidence and genotype distribution before and after national rotavirus vaccine introduction in Belgium. Vaccine. 2010;28(47):7507-13.

13. Muhsen K, Shulman L, Kasem E, Rubinstein U, Shachter J, Kremer A, et al. Effectiveness of rotavirus vaccines for prevention of rotavirus gastroenteritis-associated hospitalizations in Israel: a case-control study. Hum Vaccin. 2010;6(6):450-4.

14. Tate JE, Ngabo F, Donnen P, Gatera M, U+wimana J, Rugambwa C, et al. Effectiveness of Pentavalent Rotavirus Vaccine Under Conditions of Routine Use in Rwanda. Clin Infect Dis. 2016;62 Suppl 2:S208-12.

15. Raes M, Strens D, Kleintjens J, Biundo E, Morel T, Vyse A. Epidemiological trends for hospital admissions for acute rotavirus gastroenteritis in Belgium following the introduction of routine rotavirus vaccination and the subsequent switch from lyophilized to liquid formulation of Rotarix™. Epidemiol Infect. 2016;144(14):3017-24.

16. Hemming-Harlo M, Markkula J, Huhti L, Salminen M, Vesikari T. Decrease of Rotavirus Gastroenteritis to a Low Level Without Resurgence for Five Years After Universal RotaTeq Vaccination in Finland. Pediatr Infect Dis J. 2016;35(12):1304-8.

17. Azman AS, Parker LA, Rumunu J, Tadesse F, Grandesso F, Deng LL, et al. Effectiveness of one dose of oral cholera vaccine in response to an outbreak: a case-cohort study. Lancet Global Health. 2016;4(11):e856-e63.

18. Plenge-Bönig A, Soto-Ramírez N, Karmaus W, Petersen G, Davis S, Forster J. Breastfeeding protects against acute gastroenteritis due to rotavirus in infants. Eur J Pediatr. 2010;169(12):1471-6.

19. Paulke-Korinek M, Rendi-Wagner P, Kundi M, Kronik R, Kollaritsch H. Universal mass vaccination against rotavirus gastroenteritis: impact on hospitalization rates in austrian children. Pediatr Infect Dis J. 2010;29(4):319-23.

**Full Text Missing or no access to full text**

1. Murphy J, Cartwright E, Johnson B, Ayers T, Worthington W, Mintz ED. An evaluation of a bucket chlorination campaign during a cholera outbreak in rural Cameroon. Waterlines. 2018;37(4):266-79.

2. George CM, Monira S, Sack DA, Rashid MU, Saif-Ur-Rahman KM, Mahmud T, et al. A Randomized Controlled Trial Of A Hospital-Based Handwashing With Soap And Water Treatment Intervention (Chobi7) To Reduce Cholera Among Household Contacts Of Cholera. Am J Trop Med Hyg. 2017;95(5):184-.

3. Macdonald E, Lang S. Reducing rotaviras gastroenteritis in children. Practice Nursing. 2013;24(7):323-8.

4. García-Puebla A, García-Fragoso L. Acute rotavirus gastroenteritis in children younger than 5 years of age after initiation of rotavirus immunization schedule. Bol Asoc Med P R. 2012;104(1):4-7.

5. Xavier G. How to prevent the spread of norovirus. Nurs Times. 2011;107(2):15-7.

**Not Full Studies**

1. Cheng VC, Tai JW, Ho YY, Chan JF. Successful control of norovirus outbreak in an infirmary with the use of alcohol-based hand rub. J Hosp Infect. 2009;72(4):370-1.

2. Vinnard C, Lee I, Linkin D. Successful control of a norovirus outbreak among attendees of a hospital teaching conference. Am J Infect Control. 2012;40(1):73-4.

3. Ivers LC, Farmer PE, Pape WJ. Oral cholera vaccine and integrated cholera control in Haiti. Lancet. 2012;379(9831):2026-8.

4. Cotruvo JA. Facilitating supplemental disinfection for Legionella control in plumbing systems. J Am Water Works Assoc. 2014;106(8):74-82.

5. Atchison C, Collins S, Brown D, Ramsay ME, Ladhani S. Reduction in rotavirus disease due to the infant immunisation programme in England; evidence from national surveillance...J Infect. 2014 Jan;68 Suppl 1:S9-18. J Infect. 2015;71(1):128-31.

6. Chang MR, Velapatiño G, Campos M, Chea-Woo E, Baiocchi N, Cleary TG, et al. Rotavirus seasonal distribution and prevalence before and after the introduction of rotavirus vaccine in a peri-urban community of Lima, Peru. Am J Trop Med Hyg. 2015;92(5):986-8.

7. Kang G, Tate JE, Parashar UD. Evaluation of rotavirus disease burden and vaccine effectiveness in India. Vaccine. 2015;33(51):7143.

8. Ferreras E, Chizema-Kawesha E, Blake A, Chewe O, Mwaba J, Zulu G, et al. Single-Dose Cholera Vaccine in Response to an Outbreak in Zambia. N Engl J Med. 2018;378(6):577-9.

9. Yang TA, Hou JY, Huang YC, Chen CJ. Genetic Susceptibility to Rotavirus Gastroenteritis and Vaccine Effectiveness in Taiwanese Children. Sci Rep. 2017;7(1):6412.

10. Muhsen K, Cohen D. Rotavirus vaccines in Israel: Uptake and impact. Hum Vaccin Immunother. 2017;13(7):1722-7.

11. Travasso C. Locally run cholera intervention can reduce disease burden, finds study. BMJ (Clinical research ed). 2015;350:h2658.

12. Martinez PP, Mahmud AS, Yunus M, Faruque ASG, Ahmed T, Pascual M, et al. Tube Well Use as Protection Against Rotavirus Infection During the Monsoons in an Urban Setting. The Journal of infectious diseases. 2020;221(2):238-42.

13. Han MS, Chung SM, Kim EJ, Lee CJ, Yun KW, Choe PG, et al. Successful control of norovirus outbreak in a pediatric ward with multi-bed rooms. Am J Infect Control. 2020;48(3):297-303.p[

14. Hungerford D, Read JM, Cooke RPD, Vivancos R, Iturriza-Gómara M, Allen DJ, et al. Early impact of rotavirus vaccination in a large paediatric hospital in the UK. J Hosp Infect. 2016;93(2):117-20.

15. Redondo O, Cano R, Simón L. Decline in rotavirus hospitalizations following the first three years of vaccination in Castile-La Mancha, Spain. Hum Vaccin Immunother. 2015;11(3):769-75.

16. Yen C, Figueroa JR, Uribe ES, Carmen-Hernández LD, Tate JE, Parashar UD, et al. Monovalent rotavirus vaccine provides protection against an emerging fully heterotypic G9P[4] rotavirus strain in Mexico. J Infect Dis. 2011;204(5):783-6.

17. Martinón-Torres F, Bouzón Alejandro M, Redondo Collazo L, Sánchez Lastres JM, Pértega Díaz S, Seoane Pillado MT, et al. Effectiveness of rotavirus vaccination in Spain. Hum Vaccin. 2011;7(7):757-61.

18. Guh AY, Hadler JL. Use of the state immunization information system to assess rotavirus vaccine effectiveness in Connecticut, 2006-2008. Vaccine. 2011;29(37):6155-8.

19. Borges AM, Dias e Souza M, Fiaccadori FS, Cardoso D. Monitoring the circulation of rotavirus among children after the introduction of the Rotarix™ vaccine in Goiânia, Brazil. Mem Inst Oswaldo Cruz. 2011;106(4):499-501.

20. Becker-Dreps S, Paniagua M, Zambrana LE, Bucardo F, Hudgens MG, Weber DJ, et al. Rotavirus prevalence in the primary care setting in Nicaragua after universal infant rotavirus immunization. Am J Trop Med Hyg. 2011;85(5):957-60.

21. Boom JA, Tate JE, Sahni LC, Rench MA, Quaye O, Mijatovic-Rustempasic S, et al. Sustained protection from pentavalent rotavirus vaccination during the second year of life at a large, urban United States pediatric hospital. Pediatr Infect Dis J. 2010;29(12):1133-5.

22. Boom JA, Tate JE, Sahni LC, Rench MA, Hull JJ, Gentsch JR, et al. Effectiveness of pentavalent rotavirus vaccine in a large urban population in the United States. Pediatrics. 2010;125(2):e199-207.

23. Clark HF, Lawley D, Mallette LA, DiNubile MJ, Hodinka RL. Decline in cases of rotavirus gastroenteritis presenting to The Children's Hospital of Philadelphia after introduction of a pentavalent rotavirus vaccine. Clin Vaccine Immunol. 2009;16(3):382-6.

**No Waterborne Diarrheal Disease Outcome**

1. Zambrano LD, Priest JW, Ivan E, Rusine J, Nagel C, Kirby M, et al. Use of serologic responses against enteropathogens to assess the impact of a point-of-use water filter: A randomized controlled trial in western province, Rwanda. Am J Trop Med Hyg. 2017;97(3):876-87.

2. Loewenberg S. Treating and preventing cholera in Bangladesh. Lancet. 2017;390(10095):637-8.

3. Chen T, Gu H, Leung RKK, Liu R, Chen Q, Wu Y, et al. Evidence-Based interventions of Norovirus outbreaks in China. BMC Public Health. 2016;16(1):1-9.

4. Amador JJ, Vasquez J, Orozco M, Pedreira C, Malespin O, De Oliveira LH, et al. Rotavirus disease burden, Nicaragua 2001-2005: defining the potential impact of a rotavirus vaccination program. Int J Infect Dis. 2010;14(7):e592-5.

5. Tate JE, Panozzo CA, Payne DC, Patel MM, Cortese MM, Fowlkes AL, et al. Decline and change in seasonality of US rotavirus activity after the introduction of rotavirus vaccine. Pediatrics. 2009;124(2):465-71.

6. Assab R, Temime L. The role of hand hygiene in controlling norovirus spread in nursing homes. BMC Infect Dis. 2016;16(1).

7. Sasaki S, Suzuki H, Fujino Y, Kimura Y, Cheelo M. Impact of Drainage Networks on Cholera Outbreaks in Lusaka, Zambia. Am J Public Health. 2009;99(11):1982-7.

8. Roskosky M, Acharya B, Shakya G, Karki K, Sekine K, Bajracharya D, et al. Feasibility of a comprehensive targeted cholera intervention in the Kathmandu Valley, Nepal. Am J Trop Med Hyg. 2019;100(5):1088-97.

9. Araki K, Hara M, Shimanoe C, Nishida Y, Matsuo M, Tanaka K. Case-Control Study of Rotavirus Vaccine Effectiveness Compared to Test-Negative Controls or Hospital Controls. J Epidemiol. 2019;29(8):282-7.

10. Fiore MM, Minnings K, Fiore LD. Assessment of biosand filter performance in rural communities in southern coastal Nicaragua: an evaluation of 199 households. Rural Remote Health. 2010;10(3):1483.

11. Nsambu MN, Bazira L, Coulibaly T, Mbule A, Wilmet MD, Likwela JL. [Investigation and response to an outbreak of wild poliovirus in Kinshasa]. Pan Afr Med J. 2013;15:37.

**Waterborne Diarrheal Disease and Another Outcome**

1. Hemming M, Räsänen S, Huhti L, Paloniemi M, Salminen M, Vesikari T. Major reduction of rotavirus, but not norovirus, gastroenteritis in children seen in hospital after the introduction of RotaTeq vaccine into the National Immunization Programme in Finland. Eur J Pediatr. 2013;172(6):739-46.

2. Adlhoch C, Hoehne M, Littmann M, Marques AM, Lerche A, Dehnert M, et al. Rotavirus vaccine effectiveness and case-control study on risk factors for breakthrough infections in Germany, 2010-2011. Pediatr Infect Dis J. 2013;32(2):e82-9.

3. Du Preez M, Conroy RM, Ligondo S, Hennessy J, Elmore-Meegan M, Soita A, et al. Randomized intervention study of solar disinfection of drinking water in the prevention of dysentery in Kenyan children aged under 5 years. Environmental Science and Technology. 2011;45(21):9315-23.

4. Baker JM, Lopman BA, Tate JE, Parashar UD, Steiner CA, Haber MJ. Longer-term Direct and Indirect Effects of Infant Rotavirus Vaccination Across All Ages in the United States in 2000–2013: Analysis of a Large Hospital Discharge Data Set. Clin Infect Dis. 2019;68(6):976-83.

5. Sanneh B, Papa Sey A, Shah M, Tate J, Sonko M, Jagne S, et al. Impact of pentavalent rotavirus vaccine against severe rotavirus diarrhoea in The Gambia. Vaccine. 2018;36(47):7179-84.

6. Sáfadi MA, Berezin EN, Munford V, Almeida FJ, de Moraes JC, Pinheiro CF, et al. Hospital-based surveillance to evaluate the impact of rotavirus vaccination in São Paulo, Brazil. Pediatr Infect Dis J. 2010;29(11):1019-22.

7. Armah G, Pringle K, Enweronu-Laryea CC, Ansong D, Mwenda JM, Diamenu SK, et al. Impact and Effectiveness of Monovalent Rotavirus Vaccine Against Severe Rotavirus Diarrhea in Ghana. Clin Infect Dis. 2016;62 Suppl 2:S200-7.

8. Abeid KA, Jani B, Cortese MM, Kamugisha C, Mwenda JM, Pandu AS, et al. Monovalent Rotavirus Vaccine Effectiveness and Impact on Rotavirus Hospitalizations in Zanzibar, Tanzania: Data From the First 3 Years After Introduction. J Infect Dis. 2017;215(2):183-91.

9. Tsolenyanu E, Mwenda JM, Dagnra A, Leshem E, Godonou M, Nassoury I, et al. Early Evidence of Impact of Monovalent Rotavirus Vaccine in Togo. Clin Infect Dis. 2016;62 Suppl 2:S196-9.

10. Inchauste L, Patzi M, Halvorsen K, Solano S, Montesano R, Iñiguez V. Impact of rotavirus vaccination on child mortality, morbidity, and rotavirus-related hospitalizations in Bolivia. Int J Infect Dis. 2017;61:79-88.

11. McAuliffe GN, Taylor SL, Drinković D, Roberts SA, Wilson EM, Best EJ. Rotavirus Infection in the Auckland Region After the Implementation of Universal Infant Rotavirus Vaccination: Impact on Hospitalizations and Laboratory Implications. Pediatr Infect Dis J. 2018;37(1):e1-e5.

**Hospital acquired infections**

1. Forrest R, Jones L, Willocks L, Hardie A, Templeton K. Impact of the introduction of rotavirus vaccination on paediatric hospital admissions, Lothian, Scotland: a retrospective observational study. Arch Dis Child. 2017;102(4):323-7.

2. Illingworth E, Taborn E, Fielding D, Cheesbrough J, Diggle PJ, Orr D, et al. Is closure of entire wards necessary to control norovirus outbreaks in hospital? Comparing the effectiveness of two infection control strategies. J Hosp Infect. 2011;79(1):32-7.

3. Georgiadou SP, Loukeris D, Smilakou S, Daikos GL, Sipsas NV. Effective control of an acute gastroenteritis outbreak due to norovirus infection in a hospital ward in Athens, Greece, April 2011. Euro Surveill. 2011;16(28).

**Not clear how the effectiveness was measured**

1. McAtee CL, Webman R, Gilman RH, Mejia C, Bern C, Apaza S, et al. Burden of Norovirus and Rotavirus in Children After Rotavirus Vaccine Introduction, Cochabamba, Bolivia. Am J Trop Med Hyg. 2016;94(1):212-7.

**No Effect found/no positive outcome**

1. Matthijnssens J, Zeller M, Heylen E, De Coster S, Vercauteren J, Braeckman T, et al. Higher proportion of G2P[4] rotaviruses in vaccinated hospitalized cases compared with unvaccinated hospitalized cases, despite high vaccine effectiveness against heterotypic G2P[4] rotaviruses. Clin Microbiol Infect. 2014;20(10):O702-10.

2. Sarkar R, Ajjampur SS, Prabakaran AD, Geetha JC, Sowmyanarayanan TV, Kane A, et al. Cryptosporidiosis among children in an endemic semiurban community in southern India: does a protected drinking water source decrease infection? Clin Infect Dis. 2013;57(3):398-406.

3. Bellido-Blasco JB, Sabater-Vidal S, Salvador-Ribera Mdel M, Arnedo-Pena A, Tirado-Balaguer MD, Meseguer-Ferrer N, et al. Rotavirus vaccination effectiveness: a case-case study in the EDICS project, Castellón (Spain). Vaccine. 2012;30(52):7536-40.

4. Wobudeya E, Bachou H, Karamagi CK, Kalyango JN, Mutebi E, Wamani H. Breastfeeding and the risk of rotavirus diarrhea in hospitalized infants in Uganda: a matched case control study. BMC Pediatr. 2011;11:17.

5. Snelling TL, Andrews RM, Kirkwood CD, Culvenor S, Carapetis JR. Case-control evaluation of the effectiveness of the G1P[8] human rotavirus vaccine during an outbreak of rotavirus G2P[4] infection in central Australia. Clin Infect Dis. 2011;52(2):191-9.

**Intervention not confirmed**

1. Fu C, Dong Z, Shen J, Yang Z, Liao Y, Hu W, et al. Rotavirus Gastroenteritis Infection Among Children Vaccinated and Unvaccinated With Rotavirus Vaccine in Southern China: A Population-Based Assessment. JAMA Netw Open2018;1(4):e181382.

2. Franke MF, Jerome JG, Matias WR, Ternier R, Hilaire IJ, Harris JB, et al. Comparison of two control groups for estimation of oral cholera vaccine effectiveness using a case-control study design. Vaccine. 2017;35(43):5819-27.

3. Ali M, Debes AK, Luquero FJ, Kim DR, Park JY, Digilio L, et al. Potential for Controlling Cholera Using a Ring Vaccination Strategy: Re-analysis of Data from a Cluster-Randomized Clinical Trial. PLoS Med. 2016;13(9):e1002120.

4. Ivers LC, Hilaire IJ, Teng JE, Almazor CP, Jerome JG, Ternier R, et al. Effectiveness of reactive oral cholera vaccination in rural Haiti: a case-control study and bias-indicator analysis. The Lancet Global health. 2015;3(3):e162-8.

5. Anh DD, Lopez AL, Thiem VD, Grahek SL, Duong TN, Park JK, et al. Use of oral cholera vaccines in an outbreak in Vietnam: a case control study. PLoS Negl Trop Dis. 2011;5(1):e1006.

**Study not in real world setting e.g., measured efficacy**

1. Sévère K, Rouzier V, Anglade SB, Bertil C, Joseph P, Deroncelay A, et al. Effectiveness of Oral Cholera Vaccine in Haiti: 37-Month Follow-Up. Am J Trop Med Hyg. 2016;94(5):1136-42.

2. George CM, Monira S, Sack DA, Rashid M-u, Saif-Ur-Rahman KM, Mahmud T, et al. Randomized Controlled Trial of Hospital-Based Hygiene and Water Treatment Intervention (CHoBI7) to Reduce Cholera. Emerg Infect Dis. 2016;22(2):233-41.

3. Rashid MU, George CM, Monira S, Mahmud T, Rahman Z, Mustafiz M, et al. Chlorination of household drinking water among cholera patients' households to prevent transmission of toxigenic vibrio cholerae in Dhaka, Bangladesh: CHoBI7 trial. Am J Trop Med Hyg. 2016;95(6):1299-304.

**Outcome not clearly described and how it was confirmed**

1. Luo HM, Zhang Y, Wang XQ, Yu WZ, Wen N, Yan DM, et al. Identification and control of a poliomyelitis outbreak in Xinjiang, China. N Engl J Med. 2013;369(21):1981-90.
