# Supplementary material for: Community-level interventions for mitigating the risk of waterborne diarrheal diseases: a systematic review
Source: Syst Rev. 2022 Apr 18;11:73. doi: 10.1186/s13643-022-01947-y (PMC9016942; doi:10.1186/s13643-022-01947-y)
Supplement: Supplementary file 3 — Additional file 3: Supplementary Table 2. Characteristics of studies included in the systematic review. [file 13643_2022_1947_MOESM3_ESM.docx]

**Supplementary Table 2. Characteristics of studies included in the systematic review**

| **No** | **Title** | **Author and Year** | **Country** | **Economy** | **Disease(s)** | **Setting** | **Study Design** |
| --- | --- | --- | --- | --- | --- | --- | --- |
| 1 | Association Between Pentavalent Rotavirus Vaccine and Severe Rotavirus Diarrhoea Among Children in Nicaragua | Patel *et al*. 2009 [54] | Nicaragua | Lower middle income | Rotavirus diarrhoea | Hospitals | Case control |
| 2 | Case-control Study of the Effectiveness of Vaccination with Pentavalent Rotavirus Vaccine in Nicaragua | Mast *et al.* 2011 [52] | Nicaragua | Lower middle income | Severe wild-type rotavirus gastroenteritis | Hospitals | Case control |
| 3 | Impact of rotavirus vaccination on hospitalizations for rotavirus diarrhoea: The IVANHOE study | Gagneur *et al.* 2011 [53] | France | High income | Rotavirus diarrhoea | Hospitals | Cohort |
| 4 | Effectiveness of Pentavalent Rotavirus Vaccine Against Severe Disease | Staat *et al.*2011 [55] | USA | High income | Rotavirus acute gastroenteritis | Hospital/ medical centre | Surveillance and case control |
| 5 | Impact and Effectiveness of RotaTeq Vaccine Based on 3 Years of Surveillance Following Introduction of a Rotavirus Immunization Program in Finland | Vesikari *et al.* 2013 [50] | USA | High income | Rotavirus acute gastroenteritis | Hospitals | Surveillance and case-control |
| 6 | Impact of Rotavirus Vaccine on Premature Infants | Roué *et al.* 2014 [56] | France | High income | Rotavirus diarrhoea | Hospital | Surveillance |
| 7 | Real-World Effectiveness of Pentavalent Rotavirus Vaccine Among Bedouin and Jewish Children in Southern Israel | Leshem *et al.* 2016 [51] | Israel | High income | Rotavirus Acute Gastroenteritis | Hospital | Surveillance and Case-control |
| 8 | Effectiveness of rotavirus pentavalent vaccine under a universal immunization programme in Israel, 2011 to 2015: a case-control study | Muhsen *et al.* 2018 [48] | Israel | High income | Rotavirus gastroenteritis | Hospitals | Surveillance |
| 9 | Impact and effectiveness of pentavalent rotavirus vaccine in children <5 years of age in Burkina Faso | Bonkoungou *et al.* 2018 [49] | Burkina Faso | Low income | Rota virus acute gastroenteritis | Hospitals | Case control |
| 10 | Effectiveness of Lanzhou lamb rotavirus (LLR) vaccine in preventing gastroenteritis among children younger than 5 years of age | Li *et al.* 2019 [72] | China | Upper middle income | Rota virus diarrhoea | Hospitals | Case control |
| 11 | Effectiveness of the Lanzhou lamb rotavirus vaccine against gastroenteritis among children | Fu *et al.* 2012 [73] | China | Upper middle income | Rotavirus gastroenteritis | Hospital | Case control |
| 12 | Effectiveness of Rotavirus Vaccine in Preventing Hospitalization due to Rotavirus Gastroenteritis in Young Children in Connecticut, USA | Desai *et al.* 2010 [65] | USA | High income | Rotavirus gastroenteritis, | Hospital | Case control |
| 13 | Reduction in Paediatric Rotavirus-related Hospitalizations After Universal Rotavirus Vaccination in Belgium | Raes *et al.* 2011 [70] | Belgium | High income | Rota virus disease | Hospitals | Retrospective database |
| 14 | Effectiveness of rotavirus vaccines in preventing cases and hospitalizations due to rotavirus gastroenteritis in Navarre, Spain | Castilla *et al.* 2012 [64] | Spain | High income | Rotavirus gastroenteritis | Health Care Facilities | Case control |
| 15 | Effectiveness of Pentavalent and Monovalent Rotavirus Vaccines in Concurrent Use Among US Children <5 Years of Age, 2009–2011 | Payne *et al.* 2013 [63] | USA | High income | Rota virus acute gastroenteritis | Hospitals and medical Centres | Surveillance |
| 16 | Effectiveness of Monovalent and Pentavalent Rotavirus Vaccine | Cortese *et al.* 2013 [71] | USA | High income | Rotavirus disease | Hospitals | Surveillance and case control |
| 17 | Effectiveness of 2 Rotavirus Vaccines Against Rotavirus Disease in Taiwanese Infants | Chang *et al.* 2014 [62] | Taiwan | High income | Severe rota virus acute gastroenteritis | Hospitals | Surveillance and case control |
| 18 | Case Control Study of Rotavirus Vaccine Effectiveness in Portugal During 6 Years of Private Market Use | Marlow *et al.* 2015 [61] | Portugal | High income | Rotavirus acute gastroenteritis | Hospital | Case control |
| 19 | Long-term Consistency in Rotavirus Vaccine Protection: RV5 and RV1 Vaccine Effectiveness in US Children, 2012–2013 | Payne *et al.* 2015 [68] | USA | High income | Rota virus acute gastroenteritis | Medical facilities Centre | Surveillance |
| 20 | Association between mixed rotavirus vaccination types of infants and rotavirus acute gastroenteritis | Mohammed *et al.* 2015 [69] | USA | High income | Rotavirus acute gastroenteritis | Hospitals | Case control |
| 21 | Rotavirus vaccine effectiveness in Hong Kong children | Yeung *et al.* 2916 [58] | Hong Kong, China | Upper middle income | Acute rotavirus gastroenteritis | Hospitals | Case control |
| 22 | Sustained Effectiveness of Monovalent and Pentavalent Rotavirus Vaccines in Children | Immergluck *et al.* 2016 [59] | USA | High income | Rotavirus disease | Hospitals | Surveillance and case control |
| 23 | Effectiveness of Monovalent and Pentavalent Rotavirus Vaccines in Guatemala | Gastañaduy *et al.* 2016 [60] | Guatemala | Upper middle income | Rotavirus diarrhoea | Hospitals | Surveillance and case control |
| 24 | Rotavirus Genotypes and Vaccine Effectiveness from a Sentinel, Hospital-Based, Surveillance Study for Three Consecutive Rotavirus Seasons in Lebanon | Ali *et al.* 2016 [67] | Lebanon | Upper middle income | Rotavirus Gastroenteritis | Medical centres | Surveillance |
| 25 | Effectiveness of rotavirus vaccines against hospitalisations in Japan | Yoshiyuki *et al.* 2017 [57] | Japan | High income | Rotavirus acute gastroenteritis | Hospital | Case control |
| 26 | Effectiveness and impact of rotavirus vaccines in Saudi Arabia: A single hospital-based study | Zaki *et al.* 2017 [14] | Saudi Arabia | High income | Rotavirus gastroenteritis | Hospital | Retrospective analysis |
| 27 | Effectiveness of monovalent and pentavalent rotavirus vaccines in Japanese children | Araki *et al.* 2018 [66] | Japan | High income | Rota virus gastroenteritis | Medical facilities | Surveillance and case control |
| 28 | Effectiveness of the Monovalent G1P (8) Human Rotavirus Vaccine Against Hospitalization for Severe G2P (4) Rotavirus Gastroenteritis in Bele´m, Brazil | Justino *et al.* 2011 [40] | Brazil | Upper middle income | Severe Rotavirus Gastroenteritis | Hospitals | Case control |
| 29 | Effectiveness of rotavirus vaccination in prevention of hospital admissions for rotavirus gastroenteritis among young children in Belgium: case-control study | Braeckman *et al.* 2012 [39] | Belgium | High income | Rotavirus gastroenteritis | Hospital | Case control |
| 30 | Effectiveness of monovalent rotavirus vaccine in Bolivia: case-control study | Patel *et al.* 2013 [38] | Bolivia | Lower middle income | Rota virus | Hospital | Case control |
| 31 | Effectiveness of the monovalent rotavirus vaccine in Colombia: A case-control study | Cotes-Cantillo *et al.* 2014 [45] | Colombia | Upper middle income | Rotvirus diarrhoea | Health centres | Case control |
| 32 | Effectiveness of rotavirus vaccine against hospitalized rotavirus diarrhoea: A case–control study | Ichihara *et al.* 2014 [36] | Brazil | Upper middle income | Rotavirus diarrhoea | Hospital | Case control |
| 33 | Effectiveness of monovalent human rotavirus vaccine against admission to hospital for acute rotavirus diarrhoea in South African children: a case-control study | Groome *et al.* 2014 [37] | South Africa | Upper middle income | Acute Rotavirus diarrhoea | Hospitals | Case control |
| 34 | Effectiveness of a monovalent rotavirus vaccine in infants in Malawi after programmatic roll-out: an observational and case-control study | Bar-Zeev *et al.* 2015 [35] | Malawi | Low income | Rotavirus gastroenteritis | Hospital | Surveillance and case control |
| 35 | Effectiveness of monovalent rotavirus vaccine in a high-income, predominant use setting | Doll *et al.* 2015 [43] | Canada | High income | Rotavirus diarrhoea | Hospital | Time series analysis and case control |
| 36 | Effect of Monovalent Rotavirus Vaccine on Rotavirus Disease Burden and Circulating Rotavirus Strains Among Children in Morocco | Benhafid *et al*. 2015 [44] | Morocco | Lower middle income | Rotavirus diseases | Hospitals | Surveillance |
| 37 | Impact and Effectiveness of Monovalent Rotavirus Vaccine in Armenian Children | Sahakyan *et al.* 2016 [31] | Armenian | Upper middle income | Rotavirus gastroenteritis | Hospitals | Surveillance and case control |
| 38 | Impact of Rotavirus Vaccine Introduction and Vaccine Effectiveness in the Republic of Moldova | Gheorghita *et al.* 2016 [32] | Moldova | Lower middle income | Rotavirus diseases | Hospitals | Surveillance and case control |
| 39 | Effectiveness of Monovalent Rotavirus Vaccine After Programmatic Implementation in Botswana: A Multisite Prospective Case-Control Study | Gastañaduy *et al.* 2016 [60] | Botswana | Upper middle income | Rotavirus diarrhoea | Hospitals | Case control |
| 40 | Population Impact and Effectiveness of Monovalent Rotavirus Vaccination in Urban Malawian Children 3 Years After Vaccine Introduction: Ecological and Case-Control Analyses | Bar-Zeev *et al.* 2016 [34] | Malawi | Low income | Rotavirus diarrhoea | Hospital | Surveillance and case control |
| 41 | A Preliminary Assessment of Rotavirus Vaccine Effectiveness in Zambia | Beres *et al.* 2016 [41] | Zambia | Lower middle income | Rotavirus diarrhoea | Public health facilities | Case control |
| 42 | Effectiveness of a live oral human rotavirus vaccine after programmatic introduction in Bangladesh: A cluster-randomized trial | Zaman *et al.* 2017 [30] | Bangladesh | Lower middle income | Acute rotavirus diarrhoea | Village health care facilities | Cluster-Randomized Controlled Trial |
| 43 | Impact of rotavirus vaccination on rotavirus hospitalisation rates among a resource-limited rural population in Mbita, Western Kenya | Wandera *et al.* 2018 [28] | Kenya | Lower middle income | Rotavirus gastroenteritis | Hospital | Surveillance |
| 44 | Rotavirus gastroenteritis hospitalization rates and correlation with rotavirus vaccination coverage in Sicily | Restivo *et al.* 2018 [47] | Italy | High income | Rotavirus gastroenteritis | Hospitals | Retrospective observational |
| 45 | Sustained impact of rotavirus vaccine on rotavirus hospitalisations in Lusaka, Zambia, 2009–2016 | Mpabalwani *et al.* 2018 [29] | Zambia | Lower middle income | Rotavirus acute Gastroenteritis | Hospital | Surveillance |
| 46 | Detection of rotavirus before and after monovalent rotavirus vaccine introduction and vaccine effectiveness among children in mainland Tanzania | Jani *et al.* 2018 [42] | Tanzania | Lower middle income | Rotavirus Diarrhoea | Hospital and medical centres | Surveillance |
| 47 | Monovalent Rotavirus Vaccine Effectiveness Against Rotavirus Hospitalizations Among Children in Zimbabwe | Mujuru *et al.* 2019 [26] | Zimbabwe | Lower middle income | Rotavirus acute diarrhoea | Hospitals | Surveillance and case control |
| 48 | Rotavirus Epidemiology and Monovalent Rotavirus Vaccine Effectiveness in Australia: 2010–2017 | Maguire *et al.* 2019 [27] | Australia | High income | Rotavirus diarrhoea | Notification Centre | Case control |
| 49 | Rotavirus prevalence and seasonal distribution post vaccine introduction in Nairobi county Kenya | Gikonyo *et al.* 2019 [46] | Kenya | Lower middle income | Rotavirus diarrhoea | Hospitals | Case study |
| 50 | Description of the targeted water supply and hygiene response strategy implemented during the cholera outbreak of 2017–2018 in Kinshasa, DRC | Bompangue *et al.* 2020 [74] | Democratic Republic of Congo (DRC) | Low income | Cholera | Community, Health Zones | Preliminary community trial |
| 51 | Impact of Vi vaccination on spatial patterns of typhoid fever in the slums of Kolkata, India | Ali *et al.* 2011 [51] | India | Lower middle income | Typhoid fever | Community slum area (cluster) group of households | Cluster randomised Effectiveness trial |
| 52 | Reduction in cryptosporidiosis associated with introduction of enhanced filtration of drinking water at Loch Katrine, Scotland | Pollock *et al.* 2014 [79] | Scotland | High income | Cryptosporidiosis | Community - households | Cohort |
| 53 | Effectiveness of an oral cholera vaccine in Zanzibar: findings from a mass vaccination campaign and observational cohort study | Khatib *et al.* 2012 [78] | Zanzibar, Tanzania | Lower middle income | Cholera | Public and private treatment facilities/ households | Cohort |
| 54 | Effectiveness of an oral cholera vaccine campaign to prevent clinically significant cholera in Odisha State, India | Wierzba *et al.* 2015 [76] | India | Lower middle income | Cholera | Health care facilities | Case control and cohort |
| 55 | Long-term effectiveness of one and two doses of a killed, bivalent, whole-cell oral cholera vaccine in Haiti: an extended case-control study | Franke *et al.* 2018 [77] | Haiti | Low income | Cholera | Cholera treatment center/ household/ health facility/ household | Case control |
| 56 | Use of Vibrio cholera Vaccine in an Outbreak in Guinea | Luquero *et al.* 2014 [75] | Guinea | Low income | Cholera | Health Centres | Case control |
