# Supplementary material for: Community-level interventions for mitigating the risk of waterborne diarrheal diseases: a systematic review
Source: Syst Rev. 2022 Apr 18;11:73. doi: 10.1186/s13643-022-01947-y (PMC9016942; doi:10.1186/s13643-022-01947-y)
Supplement: Supplementary file 4 — Additional file 4: Supplementary Table 3. Objectives, participants, interventions and results of the studies included in the systematic review. [file 13643_2022_1947_MOESM4_ESM.docx]

**Supplementary Table 3. Objectives, participants, interventions and results of the studies included in the systematic review**

| **No.** | **Author** | **Objective(s)** | **Participants (sample size)** | **Control group (sample size)** | **Intervention(s); Comparison** | **Results** |
| --- | --- | --- | --- | --- | --- | --- |
| 1 | Patel *et al.* [54] | The association between Pentavalent rotavirus vaccine (RV5) vaccination and rotavirus diarrhoea requiring overnight admission or intravenous hydration in Nicaragua | Cases: Children with acute diarrhoea requiring hydration with laboratory-confirmed rotavirus (n=285), | Neighbourhood controls: children eligible to receive RV5 (n=840)  Hospital controls: children eligible to receive RV5 (n=690) | RV5; None | Three doses of RV5: Lower risk of rotavirus diarrhoea requiring overnight admission or intravenous hydration (OR 0.54; 95% CI 0.36, 0.82).  Of the 285 rotavirus cases, 191 (67%) were severe and 54 (19%) were very severe.  RV5 vaccinations lowered the risk of severe (OR 0.42; 95% CI 0.26, 0.70) and very severe rotavirus diarrhoea (OR 0.23; 95% CI 0.08, 0.61). Vaccine effectiveness: 3 doses of RV5: Hospitalisation: 46% (95% CI 18, 64); Severe rotavirus diarrhoea: 58% (95% CI 30, 74) Very severe rotavirus diarrhoea: 77% (95% CI 39, 92) |
| 2 | Mast *et al.* [52] | To evaluate the public health impact of routine universal vaccination with RV5 in Nicaragua | Children younger than five who hospitalized with acute gastroenteritis (n=502)  Vaccine effectiveness: Children, older than six weeks, positive for rotavirus, eligible to have received one dose of vaccine (n=300). | Hospital controls (n=1894) Community controls (n=1685) Vaccine effectiveness: Hospital controls (n=792) Community controls (n=851) | RV5; None | Vaccine effectiveness: 3 doses of RV5: Severe rotavirus disease: 87% (95% CI 74, 93) for community controls 64% (95% CI 44, 78) for hospital controls 76% (95% CI 63, 84) when the groups were combined |
| 3 | Gagneur *et al.* [53] | Trends in hospitalizations for rotavirus diarrhoea in infants younger than 2 years old before and after implementing a rotavirus vaccination campaign in France | Infants younger than 2 years with diarrhoea: with a positive rotavirus stool specimen (n=4798, of whom 4684 received at least one dose of RV5) | Surveillance study | RV5; None | Hospitalizations reduced by a factor of 2.04 (1.56, 2.66) during the last epidemic season (2008/2009) Relative risk for hospitalizations for rotavirus diarrhoea dropped by 98% (95% CI 83, 100) |
| 4 | Staat *et al.,* [55] | Vaccine effectiveness of complete and partial vaccination with the RV5 to prevent acute rotavirus gastroenteritis (AGE) hospitalizations and emergency department visits during the first 3 rotavirus seasons after vaccine introduction in the USA | Children older than 52 days.  Children positive for rotavirus (n=184) | Children rotavirus-negative but with AGE (n=613)  Children with Acute Respiratory Infection (ARI) (n=675) | RV5; None | Vaccine effectiveness vs AGE controls: 1 dose of RV5: 74% (95% CI 37, 90) 2 doses of RV5: 88% (95% CI 66, 96) 3 doses of RV5: 87% (95% CI 71, 94)  Vaccine effectiveness vs ARI controls: 1 dose RV5: 73% (95% CI 43, 88) 2 doses of RV5: 88% (95% CI 68, 95) 3 doses of RV5: 85% (95% CI 72, 91) |
| 5 | Vesikari *et al.* [50] | To establish the influence of RV5 vaccination and genotype on AGE cases requiring hospitalization in children from Finland | Children younger than 16 admitted for AGE  Rotavirus positive (n=127)  Cases vaccine effectiveness analysis (n=7) | Children admitted for AGE but rotavirus negative (n=73) | RV5; None | Vaccine effectiveness: 3 doses of RV5 Hospitalization: 92.1% (95% CI 50, 98.7). Hospitalizations for rotavirus AGE decreased by 78% in the post-vaccination period (2009–2012) compared to the pre-vaccination (2001–2006) period. Most cases occurred in children under 5 years |
| 6 | Roué *et al.* [56] | To establish the influence of RV5 vaccination on AGE requiring hospitalization in preterm infants enrolled in the IVANHOE study and to establish vaccine coverage and safety in this population in France | Children younger than 3 years old, born prematurely (before 37 weeks) (n=217) | Cohort study | RV5; None | Of the 217 infants, 41.9% received all three doses of the RV5 vaccine Vaccine safety in premature infants is the same as for term infants.  In the vaccinated group, hospitalisations reduced by a factor of 2.6 (95% CI 1.3, 5.2) during the first two epidemic seasons following vaccine introduction and by a factor of 11 (95% CI 3.5, 34.8) during the third season |
| 7 | Leshem *et al.* [51] | Vaccine effectiveness of RV5 vaccination in preventing rotavirus-associated Emergency Department (ED) visits and hospitalizations. To describe age, dose, ethnicity, and strain-specific vaccine effectiveness in Israel | Surveillance study: children younger than five years old  Vaccine effectiveness: children older than six months Cases: children hospitalized or visiting the ED with AGE and rotavirus positive (n=185) | Children admitted for AGE but rotavirus negative (n=330) | RV5; None | Vaccine effectiveness: 3 doses of RV5: 63% (95% CI 38, 78) Age 6-11 months: 64% (95% CI 21, 84) 12-23 months: 71% (95% CI 39, 86) Hospitalization: 59% (95% CI 23,78) ED visit: 67% (95% CI 11, 88) |
| 8 | Muhsen *et al.* [48] | Effectiveness of the RV5 universal vaccination programme in preventing rotavirus AGE hospitalization between 2011 and 2015 in Israel, a high-income country | Children (0 to 59 months) hospitalized due to diarrhoea. Cases: children at least 2 months of age and rotavirus positive (n=98) | Children admitted for AGE but rotavirus negative (n=628) | RV5; None | Vaccine effectiveness: 3 doses of RV5 Age 6-59 months: 77% (95% CI 49, 90) 6-23 months: 86% (95% CI 65, 94) Incomplete schedule: Age 6-59 months: 72% (95% CI 28, 89) 6-23 months: 75% (95% CI 30, 91) Genotypes G1P [8]:79% (95% CI 45,92) RVGE: 69% (95% CI 11,89) |
| 9 | Bonkoungou *et al.* [49] | Effectiveness of the RV5 vaccine in Burkina Faso | Children younger than 5 (n=1043) Cases: children at least 6 months old, eligible to have received rotavirus vaccine, rotavirus positive (n=227) | Same age as cases and rotavirus negative (n=761) | RV5; None | Reduced hospital admissions positive for rotavirus: 2014: 36% (154/422), 2015: 22% (71/323), 2016: 20% (61/298) Reduced hospital admissions of infants with rotavirus 2014: 38% (94/250), 2015: 21% (32/153), 2016: 17% (26/149) Vaccine effectiveness: 3 doses of RV5: Age: 6-11months: 58% (95% CI 10, 81) Older than 12 months: 19% (95% CI 78, 63) |
| 10 | Li *et al.* [72] | Vaccine effectiveness of the Lamb Rotavirus (LLR) vaccine to prevent laboratory-confirmed gastroenteritis in children 2–59 months of age in China | Children 2 to 59 months old Cases: rotavirus positive (n=598) | Same aged children: rotavirus negative (n=1766) | LLR vaccine; None | Vaccine effectiveness: 1 dose of LLR vs. no vaccine: 34.9% (95% CI 5.3, 55.3) Severe gastroenteritis: 87.7% (95% CI 32.7, 97.8) Age: 2-35 months: 36.2% (95% CI 4.7, 57.3) Genotype G9: 40.8% (95% CI 7.8, 61.9) Compared to unvaccinated children, vaccinated children were less likely to have watery stool (OR = 0.42) and have diarrhoea longer than 5 days (OR = 0.47) |
| 11 | Fu *et al.* [73] | Effectiveness of the LLR prevent rotavirus gastroenteritis in children 2–35 months of age | Children 2–35 months old with watery vomiting and watery diarrhoea. Cases: rotavirus positive (n=3130) | Randomly selected children aged 2–35 months without gastroenteritis (n=3607) | LLR vaccine; None | Vaccine effectiveness: 1 dose of LLR: Age:9–11 months old: 44.3% (95% CI 28.4, 56.7) 12-17 months old: 52.8% (95% CI 40.8, 62.3) 18-35 months old: 51.8% (95% CI 11.6, 73.8) |
| 2 | Desai *et al.* [65] | Effectiveness of rotavirus vaccines in preventing hospitalization due to rotavirus in children 8 weeks to 3 years of age | Children 8 weeks to 3 years old Cases: children hospitalised for AGE, rotavirus positive (n=42) | Controls: age matched; rotavirus negative Hospital controls (n=80) Community controls (n=73) | RV1 & RV5; None | Vaccine effectiveness: At least 1 dose Cases vs. hospitalized controls: 94.3% (95% CI 55.4, 99.3; p=0.006)  Cases vs. community controls: 96.9% (59.4, 99.8; p=0.008)  Partial vaccination: Cases vs. hospitalized controls: 93.2% (41.4, 99.2) Cases vs. community controls: 93.8% (23, 99.5)  Full vaccination: Cases vs. hospitalized controls: 96.3% (28.9, 99.8) Cases vs. community controls 99.1% (78.1, 99.9) |
| 13 | Raes *et al.* [70] | Effect of rotavirus vaccines on rotavirus-hospitalizations in children younger than 5 years old before and after the introduction of generalized vaccination in Belgium | Children less than or equal to 5 years old, hospitalised, rotavirus positive  In Pre-vaccination (June 2004–May 2006) and postvaccination  (June 2007–May 2009) periods | None | RV1 & RV5; None | Rotavirus hospitalizations declined among children 2 to 24 months from 716 (pre-vaccine) to 249 (post-vaccine).  Hospitalizations declined by 65% (95% CI 62, 69) in the first year. Hospitalizations further decreased by 140 (80%, 95% CI 77, 83) in the second year post vaccination. Age Younger than 2 months: 1^st^ year post-vaccine: hospitalizations declined by 50% (95% CI 36, 64) 2^nd^ year post-vaccine: hospitalizations declined by 64% (95% CI 49, 76) Older than 24 months: 1^st^ year post-vaccine: hospitalizations declined by 20% (95% CI 14, 28) 2^nd^ year post-vaccine: hospitalizations declined by 64% (95% CI 56, 72) |
| 14 | Castilla *et al.* [64] | Effectiveness of rotavirus vaccination in children 3–59 months old in preventing rotavirus AGE and hospital admissions | Children 3–59 months seeking medical care for AGE Cases: rotavirus positive (n=756) | Children seeking medical care for AGE but rotavirus negative (n=6036) | RV1 & RV2; None | Vaccine effectiveness  At least one dose: Age 3-59 months: 78% (95% CI 70, 84) RV1: 76% (95% CI, 63, 85) RV5: 80% (95% CI 69, 87) Complete vaccination vs. not vaccinated: 78% (95% CI 68, 85) 2 doses of RV1: 75% (95% CI 60, 85) 3 doses of RV5: 81% (95% CI 68, 89) Age >24 months: 61%; 95% CI 0, 84) Age <24 months: 80%; 95% CI 70, 86). Preventing hospitalizations: 83% (95% CI 65, 93) Preventing outpatient cases: 75% (95% CI: 62, 83) |
| 15 | Payne *et al.* [63] | To assess RV5 and RV1 vaccine effectiveness in preventing rotavirus AGE hospitalization and emergency department (ED) visits among US children <5 years of age over 2 consecutive rotavirus seasons in USA | Children younger than 5 years old with AGE. Either hospitalised or in the ED Cases: rotavirus positive (n=359) | Children with AGE, rotavirus negative (n=1811) | RV1 & RV5; None | Vaccine effectiveness  3 doses of RV5: 84% (95% CI 78, 88) 2 doses of RV1: 70% (95% CI 39, 86) |
| 16 | Cortese *et al.* [71] | Vaccine effectiveness of the 2-dose RV1 and 3-doses RV5 series against rotavirus disease resulting in hospitalization, emergency department or inpatient care USA | Children older than 8 months presenting to the hospital with AGE Cases: rotavirus positive (n=165) | Children older than 8 months presenting to the hospital with AGE, rotavirus negative (n=428) Community controls (n=5489) | RV1 & RV2; None | Vaccine effectiveness  Age >8 months RV1: 91% (95% CI 80, 95) RV5 92% (95% CI 75, 97)  Age 12-23 months RV1: 91% (95% CI 75, 96) Genotypes RV1_G2P 94% (95% CI 78, 98) RV1_G1P 89% (95% CI 70, 96). |
| 17 | Chang *et al.* [62] | Vaccine effectiveness of RV1 and RV5 against rotavirus AGE resulting in hospitalization among children in Taiwan | Children 8–35 months hospitalized with AGE; Cases: rotavirus positive (n=184) | Hospital controls: non-AGE, rotavirus negative (n=909) AGE, rotavirus negative (n=904) | RV & RV5; None | Vaccine effectiveness  2 doses of RV1: vs. RV-negative AGE: 90.4% (95% CI 70.3, 98.1) vs. RV-negative non-AGE: 92.5% (95% CI 77.1, 98.5)  3 doses of RV5: vs. RV-negative AGE: 96.8% (95% CI 82.3, 100) vs. RV-negative non-AGE: 97.1% (95% CI 84, 100) |
| 18 | Marlow *et al.* [61] | Vaccine effectiveness in Coimbra: a low vaccine coverage setting Portugal | Children 8 weeks ≤36 months, with AGE  Cases: rotavirus positive (n=542) | Children 8 weeks ≤36 months, with AGE  Cases: rotavirus negative (n=1099) | RV1 & RV5; None | Vaccine effectiveness  At least 1 dose of either RV1 or RV5 against AGE: 83.7% (95% CI 73.9, 89.8) against hospital admission: 96.1% (95% CI 83.8, 99.1)  Full course of either RV1 or RV5 against attendance: 83% (95% CI 71.8, 89.7) against admission: 97.5% (95% CI 81.4, 99.7) |
| 19 | Payne *et al.* [68] | Vaccine effectiveness of RV5 and RV1 in preventing rotavirus AGE hospitalization and emergency department (ED) visits among US children during 2 rotavirus seasons (2012 and 2013) | Children younger than 8 years Cases: rotavirus positive, RV5 (n=402), RV1 (n=100) | Children younger than 8 years, rotavirus negative, RV5 (n=2559), RV1 (n=804) | RV5 & RV1; None | Vaccine effectiveness  3 doses of RV5: 80% (95% CI 74, 84) 2 doses of RV1: 80% (95% CI 68, 88) |
| 20 | Mohammed *et al.* [69] | Vaccine effectiveness of RV1 and RV5: incomplete, complete and mixed regimens against rotavirus infection, association with severity of disease USA | Children born after March 1, 2009, presenting with AGE Cases: rotavirus positive (n=215) | Children born after March 1, 2009, presenting with AGE Cases: rotavirus negative (n=493) | RV1 & RV5; None | Children >12 months: more likely to have rotavirus Severity score >11, twice as likely to be rotavirus positive. Prior rotavirus vaccination decreased the mean Vesikari score, p < 0.0001. Complete vaccination with either RV1 or RV5, protected (OR: 0.21, 95% CI 0.14, 0.31, p < 0.0001) |
| 21 | Yeung et al.[58] | Vaccine effectiveness of RV1 and RV1 in preventing rotavirus AGE in Hong Kong, China | Children, 1 month to 5 years, Cases: rotavirus positive within 48 hours of hospitalisation (n=126) | Children, 1 month to 5 years, rotavirus negative (n=278) | RV1 & RV5; None | Vaccine effectiveness  At least 1 dose of either vaccine: 92% (95% CI 75, 98). Age matched: 96% (95% CI 72, 100) Age and admission date matched: 89% (95% CI 51, 97) |
| 22 | Immergluck *et al.* [59] | Vaccine effectiveness of the RV1 and RV5 vaccines USA | Children, ≥8 months, presenting to the ED with AGE Cases: rotavirus positive (n=98) | Children, ≥8 months, presenting to the ED with AGE Cases: rotavirus negative (n=175) | RV1 & RV5; None | Vaccine effectiveness  Age 8-23 months 2 doses of RV1: 84% (95% CI 38, 96) 3 doses of RV5: 80% (95% CI 27, 95) Age >24 months 2 doses of RV1: 82% (95% CI 41, 95) among 3 doses of RV5: 87% (95% CI 22, 98) |
| 23 | Gastañaduy *et al.* [60] | Vaccine effectiveness of RV1 and RV5 against rotavirus diarrhoea requiring emergency department (ED) care or hospitalization in Guatemala | Vaccine eligible children (born after June 2009), presenting to the ED or hospital with AGE Cases: rotavirus positive (n=213) | Two control groups: children, non-AGE, rotavirus negative (n=657)  Children with AGE, rotavirus negative (n=334) | RV1 & RV5; None | Vaccine effectiveness  2 to 3 doses of either RV1 or RV5 vs. hospital controls: 74% (95% CI 58, 84) vs. test-negative controls: 52% (95% CI 26, 69)  Vaccine effectiveness was similar across age groups and vaccine type |
| 24 | Ali *et al.* [67] | Surveillance study: Rotavirus infections in a Lebanese paediatric population, younger than five years | Children, <5 years, admitted for AGE Cases: rotavirus positive (n=428) | Children, <5 years, admitted for AGE Cases: rotavirus negative (n=986) | RV1 & RV5; None | Median duration of hospitalization was 4 days.  RV negative subjects were more likely to be RV vaccinated (21%) compared to the RV positive subjects (11.3%) (P<0.001), vaccine breakthrough rate of 18.8%.  RV1: RV5 (7.8:1) Vaccine effectiveness  RV1 and RV5: 68.4% (95% CI 49.6, 80.2) |
| 25 | Yoshiyuki *et al.* [57] | Vaccine effectiveness of RV1 and RV5 against rotavirus hospitalisation in northern Japan, where there is a burden of rotavirus disease | Children, 8-59 weeks, with AGE Cases: rotavirus positive (n=55) | Children, 8-59 weeks, with AGE, rotavirus negative (n=189) | RV1 or RV5; None | Vaccine effectiveness  Either RV1 or RV5: 70.4% (95% CI 36.0, 86.4) |
| 26 | Zaki *et al.* [14] | Vaccine effectiveness of rotavirus vaccines in reducing hospitalisations for rotavirus AGE in Saudi Arabia, before and after rotavirus vaccines were added to the national vaccination schedule | Two groups (n=730): Group 1: patients admitted to hospital 1 year before the vaccine was introduced to the national vaccination schedule Group 2: patients admitted to the hospital 3 years post-vaccine | None | RV1 & RV5; None | Prevalence of rotavirus-positive gastroenteritis dropped from 38.5% in group 1 to 13.2% in group 2 (P = 0.0001). Median age of rotavirus infection (P = 0.003) Pre-vaccine period: 16 (95% CI 12, 36) months post-vaccine period: 44 (95% CI 21, 56) |
| 27 | Araki *et al.* [66] | Vaccine effectiveness and duration of protection of RV1 and RV5 against rotavirus AGE, RVGE severity and RV genotype among children aged <3 years in Japan | Children, ≥ 2 months to <3 years, presenting with AGE Cases: rotavirus positive (n=487) | Children, ≥ 2 months to <3 years, presenting with AGE, rotavirus negative (n=925) | RV1 or RV5; None | Vaccine effectiveness  against rotavirus AGE: 80.0% (95% CI 72.8, 85.5) RV1: 80.6% (95% CI 70.7, 87.1) RV5: 80.4% (95% CI 69.1, 87.6) Duration of protection: against AGE >70% up to 2 years after vaccination Vaccine effectiveness increased with severity of AGE: 97.3% (95% CI 88.8, 99.3). RV1 and RV5 similar effectiveness against G1P [8] and G2P[4] |
| 28 | Justino *et al.* [40] | Vaccine effectiveness (VE) against severe rotavirus gastroenteritis (RVGE)  hospitalizations in Brazil | Children, > 12 weeks, hospitalized with AGE Cases: rotavirus positive (n=538) | Hospital controls: no AGE, children matched to cases by date of birth (n=507) Neighbourhood controls: children, no signs of gastroenteritis, from the same neighbourhood as the case (n=346) | RV1; None | Vaccine effectiveness  against rotavirus AGE vs. neighbourhood controls: 75.8% (95% CI 58.1, 86.0)  Age 3 to 11 months 95.7% (95% CI, 67.8, 99.4) Age >12months 65.1% (95% CI, 37.2, 80.6) vs. hospital controls: 40.0% (95% CI 14.2, 58.1) Age 3 to 11 months: 55.6% (95% CI, 12.3, 77.5)  Age >12 months: 32.1% (95% CI 3.7, 55.5) Genotypes G2P [4]: 82.0% of AGE hospitalizations. Vaccine effectiveness for G2P [4] vs. neighbourhood controls: 75.4% (95% CI 56.7, 86.0) vs. hospital controls: 38.9% (95% CI: 11.1, 58.0) |
| 29 | Braeckman *et al.* [39] | Vaccine effectiveness in preventing admission to hospital for rotavirus AGE among young children in Belgium. The study also assessed the burden of rotavirus disease, distribution of rotavirus genotypes, and co-infections with other common intestinal viruses | Children who had received at least one dose of any rotavirus vaccine, > 14 weeks, presenting with AGE Cases: rotavirus positive (n=215) | Children matched to cases by date of birth, not admitted for AGE, rotavirus negative (n=276) | RV1; None | Vaccine effectiveness: 2 doses of RV1 against AGE: 90% (95% CI 81, 95) against admission: 90% (95% CI 79, 96) against severe rotavirus: 91% (80% to 96%) against moderate to mild: 66% (−31% to 91%) against co-infections (adenovirus, astrovirus and/or norovirus): 86% (95% CI 52, 96) at least 1 dose of RV1 against admission: 91% (95% CI 82, 95).  Genotypes against G2P[4]: 85% (95% CI 64, 94) against G1P [8] 95% (95% CI 78, 99) |
| 30 | Patel *et al.* [38] | Vaccine effectiveness of 2 doses of RV1 against hospital admissions for rotavirus in Bolivia | Children, > 8 weeks, presenting with AGE Cases: rotavirus positive (n=400) | Hospital controls Children, > 8 weeks, presenting with AGE, rotavirus negative (n=718)  Children, not with AGE (n=1200) | RV1; None | Vaccine effectiveness  RV1 against hospital admission: vs. rotavirus negative controls: 69% (95% CI 54, 79) vs. non-diarrhoea controls: 77% (95% CI 65, 84) one dose of RV1 vs. rotavirus negative controls: 36% vs. non-diarrhoea controls: 56%  Protection was sustained through two years of life Hospital admissions: children under 1 year (64% and 77%) and over 1 year of age (72% and 76%). RV1 provided significant protection against diverse serotypes of Rotavirus |
| 31 | Cotes-Cantillo *et al.* [45] | Vaccine effectiveness of RV1 in preventing rotavirus AGE admissions to emergency departments (ED) in Colombia | Children, > 8 weeks, presenting with AGE Cases: rotavirus positive (n=193) | Children, > 8 weeks, presenting with AGE, rotavirus negative (n=858) | RV1; None | Vaccine effectiveness of RV1 Age 6-11 months 79.19% (95% CI 23.7, 94.32)  Age >12 months: −39.75% (95% CI −270.67, 47.24)  Against overnight hospitalizations: Age 6-11 months: 84.42% (95% CI 22.68, 96.86)  Age >12 months: −79.49% (95% CI, −555.8 to 51.08) |
| 32 | Ichihara *et al.* [36] | Vaccine effectiveness of RV1 in preventing hospitalization for rotavirus AGE in Brazil and genotype-specific vaccine effectiveness by time since second vaccine dose | Children, 4 to 24 months, with AGE Cases: rotavirus positive (n=215) | Children rotavirus negative matched by age and sex to cases (n=1961) | RV1; None | Vaccine effectiveness  2 doses of RV1: 76% (95% CI 58, 86) lasting for two years after adjusting for confounders: 72% (95% CI 44, 85) Genotypes against G1P [8]: 89% (95% CI 78, 95) against G2P [4]: 76% (95% CI 64, 84) against all G1: 74% (95% CI 35, 90) against all G2: 76% (95% CI 63, 84) all non G1/G2 genotypes: 63% (95% CI 27, 99).  1 dose of RV1: 62% (95% CI 39, 97). |
| 33 | Groome *et al.* [37] | Vaccine effectiveness of RV 1 in preventing admission for rotavirus AGE in children younger than 2 years in a high HIV setting, where infants are HIV exposed in South Africa | Children, 18 weeks to 23 months, admitted with AGE Cases: rotavirus positive (n=540) | Children, 18 weeks to 23 months, admitted with AGE and respiratory infection, rotavirus negative (n=1434) | RV1; None | Vaccine effectiveness  2 doses of RV1 vs rotavirus-negative controls: 57% (95% CI 40, 68) (similar to respiratory controls) Age 12-23 months: 60% (95% CI 21, 80) Age: 18 weeks -22 months: 66% (95% CI 46, 79) Age: 18 weeks to 23 months: 63%(95% CI 45, 75) Adjusted: 18 weeks-11 months: 54% (95% CI 32, 68) Adjusted: 12-23 months: 61% (95% CI 35, 77) HIV exposed: 64% (95% CI 34, 80) HIV unexposed: 54% (95% CI 31, 69) 1 dose of RV1 vs. rotavirus negative controls: 40% (95% CI 16, 57) (similar to respiratory controls)  Age 12-23 months: 41% (95% CI -17, 71) Age: 18 weeks -22 months: 60% (95% CI 34 to 76) Age: 18 weeks to 23 months: 54% (95% CI 31 to 69) |
| 34 | Bar-Zeev *et al.* [35] | Vaccine effectiveness of a complete series of RV1 against rotavirus diarrhoea hospitalisation in Malawi | Children, <5years, presenting with AGE Cases: rotavirus positive (n=118) | Hospital controls: presenting with AGE, rotavirus negative (n=317) Community controls (n=380) | RV1; None | Pre-vaccination program: 79/157 (50%) rotavirus positive 2 years Post-vaccination program: 52/170 (31%) rotavirus positive  Incidence of hospital admission: 2012: 269/ 100 000; 2013: 284/100 00; 2014: 153/ 100 000, incidence dropped by 43.2% (95% CI 18, 60.7) Vaccine effectiveness of 2 doses of RV1:  vs. rotavirus-negative individuals: 64% (95% CI 24, 83) vs. community controls: 63% (95% CI 23, 83) Vaccine was more effective against genotype G1 than against G2 and G12 |
| 35 | Doll *et al.* [43] | Vaccine effectiveness of RV1 in preventing rotavirus emergency visits and hospitalizations among young children in Canada, examine the effect of increasing vaccination coverage on the prevalence of paediatric rotavirus over time | Children, 8 weeks to <3 years, presenting with AGE, diarrhoea, vomiting Cases: rotavirus positive (n=32) | Children, 8 weeks to <3 years, presenting with AGE, diarrhoea, vomiting, rotavirus negative: (n=342) Surveillance (n=866) | RV1; None | 2012-13 season vs. 2013-14 season: reduced prevalence of 70.1% (95% CI 21.9, 88.6)  A 1% increase in 2 dose RV1 coverage in children 1 year of age: reduced prevalence of 3.8% (95% CI 1.8, 5.8) Reduced prevalence of rotavirus homotypic strain: 2011–12 season: 77% (95% CI 68, 89) vs. 2013–14 season: 8% (95% CI 0, 36)  Vaccine effectiveness of 2 dose RV1: 91.2% (95% CI 61.6, 98.0) |
| 36 | Benhafid *et al.* [44] | Vaccine effectiveness of RV1 in reducing the prevalence of children hospitalized with rotavirus diarrhoea; describe rotavirus genotype and prevalence before and after the introduction of vaccination in Morocco | Children, < 5 years, diarrhoea positive,  Diarrhoea positive Pre-vaccine: (n=1861) post-vaccine: (n=533)  Rotavirus positive cases  Pre-vaccine: Cases (n=766) Post-vaccine: Cases (n=128) | None | RV1; None | Pre-vaccine (2006 to 2010): 1861 children hospitalized with AGE; 766 (41%) rotaviruses positive. post-vaccine (2011 to 2013): 533 children hospitalized with AGE, 128 (24%) rotavirus positive.  Overall decline in prevalence of 41.5% |
| 37 | Sahakyan *et al.* [31] | Vaccine effectiveness of RV1 on reducing disease burden in Armenia | Children, 0–59 months, with diarrhoea. Cases: rotavirus positive, between 6 months and 2 years old | Children, 0–59 months, with diarrhoea, rotavirus negative | RV1; None | 1st year post-vaccination: 48% reduction in rotavirus hospitalizations in infants 2^nd^ & 3^rd^ years post-vaccinations: ≥75% reduction in rotavirus hospitalizations in infants Hospitalisations reduced by ≥30% in non-vaccinated children in 3^rd^ year post-vaccine introduction Hospitalizations reduced 69% in children aged <5 years. Vaccine effectiveness of 2 doses of RV1 against hospitalization: Age 6–23 months: 62% (95% CI 36, 77) Aged 6–11 months: 68% (95% CI 24, 86) Age 12-23 months: 60% (95% CI 20, 80) against severe rotavirus disease: 79% (95% CI 55, 90) |
| 38 | Gheorghita *et al.* [32] | Vaccine effectiveness of RV1 in Moldova, impact of RV1 introduction on rotavirus-associated hospitalizations in Moldova | Children, younger than 5 years, presenting with AGE Cases: rotavirus positive, older than 6 months (n=100) | Children, younger than 5 years, presenting with AGE, rotavirus negative (n=875) | RV1; None | Hospitalization for positive rotavirus declined, also in unvaccinated children: 1^st^ year post-vaccine: 45% to 25% (rate reduction, 36%; 95% CI 26, 44) 2^nd^ year post-vaccine: 14% (rate reduction, 67%; 95% CI 48, 88) The highest reduction in hospitalizations of infants younger than 1 year old. Vaccine effectiveness of 2 doses of RV1: against hospitalization: 79% (95% CI 62, 88) against severe disease: 84% (95% CI, 64, 83) |
| 39 | Gastañaduy *et al.* [60] | Vaccine effectiveness of RV1 in preventing rotavirus diarrhoea requiring hospitalization in Botswana | Children, ≥ 4 months hospitalised with diarrhoea and/or vomiting Cases: rotavirus positive (n=242) | Children, ≥4 months, hospitalised with diarrhoea and/or vomiting, rotavirus negative (n=368) | RV1; None | Vaccine effectiveness of 2 doses of RV1 against hospitalization: 54% (95% CI 23, 73) 1 dose of RV1: 48% (95% CI 1, 72) against G2P [4]: 59% (95% CI 4, 83) Nutrition status & 2 doses of RV1: no undernutrition: 75% (95% CI 41, 89) moderate or severe undernutrition: -28% (95% CI -309% to 60%) (P = 0.02) |
| 40 | Bar-Zeev *et al.* [34] | Vaccine effectiveness of RV1 and rotavirus prevalence in diarrheal stool and hospitalization incidence before and after rotavirus vaccine introduction in Malawi | Children, <5 years, presenting with AGE, HIV positive or exposed or stunted. Cases: rotavirus positive (n=241) | Children, <5 years, presenting with AGE, HIV positive or exposed or stunted, rotavirus negative (n=692) | RV1; None | Vaccine effectiveness  Age <12 months: 70.6% (95% CI 33.6, 87.0) Age 12 to 24 months: 31.7% (95% CI −140.6, 80.6) Vaccine effectiveness not influenced by nutritional status (P=0.12) or HIV exposure (P=0.91) well nourished: 78.1% (95% CI 5.6, 94.9) in stunted: 27.8% (95% CI -99.5, 73.9) HIV unexposed: 60.5% (95% CI 13.3, 82.0) HIV exposed: 42.2% (95% CI, −106.9, 83.8) |
| 41 | Beres *et al.* [41] | Vaccine effectiveness of the RV1 vaccine in Lusaka province Zambia | Children, 0 to 59 months, presenting with diarrhoea Cases: rotavirus positive (n=125) | Children, 0 to 59 months, presenting with diarrhoea, rotavirus negative (n=404) Surveillance, children with vaccination cards (n=1506) | RV1; None or pentavalent vaccine (DTP-Hib-HepB) | Vaccine effectiveness of 2 doses of RV1: Age >6months: 26% (95% CI, −30, 58) against hospitalization: 56% (95% CI, −34, 86) |
| 42 | Zaman *et al.* [30] | Effectiveness of a rotavirus vaccination program in reducing the risk of presenting with rotavirus AGE in vaccination eligible children in Bangladesh | Children, <2 years, eligible for vaccination Cases: 72 villages in vaccine areas with 6527 eligible children | 72 villages in non-vaccine areas with 5791 eligible children | RV1; None | Incidence of rotavirus AGE non-vaccine villages: 4.10/ 100 person-years vaccine villages: 2.8/ 100 100 person-years Overall effectiveness: 29.0% (95% CI 11.3, 43.1) |
| 43 | Wandera *et al.* [28] | Effectiveness of a rotavirus vaccination program in Western Kenya on rotavirus AGE and distribution of strains | Children, <5 years Cases: rotavirus positive (n=323) | None | RV1; None | Hospitalizations due to rotavirus AGE declined by 48% (95% CI 27, 64) 1^st^ year decline: 40% 2^nd^ year decline: 51% Dominant strain changed from GIP (8) to G2P (4) after the introduction of the vaccine |
| 44 | Restivo *et al.* [47] | Impact of vaccination coverage on rotavirus AGE hospitalization rates in Sicily Italy, after the first five-years universal rotavirus vaccination, to evaluate changes in hospitalization in different age-groups and Provinces | Children, 0 to 59 months Pre-vaccination (2009 - 2012) Post vaccination (2013 - 2017) | None | RV1; None | Pre-vaccination hospitalizations: 394/ 100 000 post-vaccination hospitalizations: 200/ 100 000 49.2% overall reduction in hospitalizations. Reductions in hospitalization by age: Age 0 to 11 months (-61.4%) Age 12-23 months (-51.2%) Age 24-35 months (-48.8%) |
| 45 | Mpabalwani *et al.* [29] | Impact of rotavirus vaccine on hospitalisations for rotavirus AGE in children <5 years in the first four years after introducing the vaccine in Zambia | Children, <5 years, hospitalised for AGE Pre-vaccine: Jan 2009-Dec 2011 Post-vaccine: Jan 2013-Dec 2016 | None | RV1; None | Pre-vaccine hospitalizations: 40% Post-vaccine hospitalizations: 29%  Significant reduction (p < 0.001)  Rotavirus positivity decreased from 2013 to 2015, and increased to 37% in 2016.  Post-vaccine years (2012 to 2016), fewer tests conducted (median decline: 34% range: 20 to 43) and lower positivity (median decline: 52% range: 30 to 65) |
| 46 | Jani *et al.* [42] | Vaccine effectiveness of RV in preventing hospitalisations, and impact on detection rate before and after the vaccine was introduced in mainland Tanzania | Children, < 5 years, presenting with AGE Cases: rotavirus positive (n=154) | Children, < 5 years, presenting with AGE, rotavirus negative (n=670) | RV1; None | Positivity declined after the vaccine was introduced Vaccine efficacy of >/1 RV1 dose against hospitalization among children 5 to 23 months: 53% (95% CI -14, 81) against hospitalization with intravenous rehydration: 66% (95% CI 9, 87) |
| 47 | Mujuru *et al.* [26] | Vaccine effectiveness of routine RV1 vaccination in Zimbabwe | Children, > 6 months, < 5 years Cases: rotavirus positive (n=903) | Children, > 6 months, < 5 years Cases: rotavirus negative (n=2685) | RV1; None | Vaccine effectiveness of 2 doses of RV1: against hospitalization, any severity: 61% (95% CI 21, 81) against severe disease: 68% (95% CI 13, 88) Nutritional status stunted infants: 45% (95% CI -148, 88) normal height for age: 71% (95% CI 29, 88) |
| 48 | Maguire *et al.* [27] | Vaccine effectiveness of RV1, surveillance of rotavirus disease, epidemiology, and genotypic profiles in Australia | Children, > 6 months eligible for vaccination. Cases: rotavirus positive (n=3587), 2010-2017 | Cased matched controls 10 controls for each case | RV1; None | Vaccine effectiveness of 2 doses of RV1:  Age 6 to 11 months: 88.6% Age 1 to 3 years: 83.7% Age: 4 to 9 years: 78.7%  1^st^ year of vaccination:  5^th^ to 10^th^ year post-vaccination: 77.05% Equine like G3P [8] (48%) and G8P [8] (23%) were most common genotypes in case patients >/6 months. |
| 49 | Gikonyo *et al.* [46] | Impact of RV1 vaccination on the prevalence, age and seasonal distribution of rotavirus gastroenteritis in urban, Nairobi County, Kenya | Children, < 5 years Cases: rotavirus positive (n=323), 2015-2017 | None | RV1; None | Rotavirus infections detected in 49/323 faecal samples, prevalence = 15.2%. In 2015, 21/95 (22.1%) samples were rotavirus positive. In 2016, 17/115 (14.8%) samples were rotavirus positive. In 2017, 11/ 113 (10%) samples were rotavirus positive. Age distribution of rotavirus prevalence  ≤ 6 months: 8.5%, 7 to 12 months: 27.4%, 13 to 24 months: 41.4%, 25 to 36 months: 16.4%, 36 to 65: 6.3%.  Rotavirus diarrhoea was more common in wet and cold months, highest prevalence in August (24.5%), July and March (12.3%), April (10.2%). |
| 50 | Bompangue *et al.* [74] | Temporal pattern of cholera outbreaks, number of cases per health zone in Kinshasa Province DRC, before and after interventions were implemented | Any person >/2 years, cholera cases (2017 to 2018), n=1712 | None | Grid approach: Emergency water supply, Household water treatment, safe storage, home disinfection, hygiene promotion; None | Weekly cholera cases dropped by an average of 57% after 2 weeks and 86% after 4 weeks of interventions. The total weekly cases dropped by 71% , following the peak of the outbreak. |
| 51 | Ali *et al.* [80] | The impact of the mass vaccination campaign on the spatial patterns of typhoid fever using Geographic Information System (GIS) methodologies | Persons aged two years and older, living in the area where Vi vaccine was given (n=37763) 1^st^ year post-vaccine (n=37 578) 2^nd^ year post vaccine (n=36 376) | Persons who got a single dose of the inactivated hepatitis A vaccine, outside of the Vi vaccine area | Vi polysaccharide; Inactivated hepatitis A vaccine | Typhoid was randomly distributed in the pre-vaccine period. Following mass vaccination, control clusters were the high-risk areas for typhoid, low-risk areas were dominated by Vi clusters. Control clusters surrounded by Vi clusters also had low risk for typhoid fevers. Pre-vaccination incidences:  Salmonella typhi (S. typhi): 194/100 000 Salmonella paratyphi (S. paratyphi): 104/100 000  1^st^ year post-vaccination: incidence of S. typhi dropped, incidence of S. paratyphi similar to pre-vaccination period. 2^nd^ year post-vaccination period:  S. typhi: 190/100 000 S. paratyphi: 170/100 000 |
| 52 | Pollock *et al.* [79] | Association between Loch Katrine sourced water with the local incidence of cryptosporidiosis in Scotland | Community members with cryptosporidiosis (n=395) | None | Enhanced water filtration/ No enhanced filtration/ non-Loch Katrine areas | Incidence of cryptosporidiosis associated with, unfiltered Loch Katrine drinking water supplied to the home (OR, 1.86, 95% CI 1.11, 3.11) |
| 53 | Khatib *et al.* [78] | Effectiveness of oral cholera vaccination in high-risk populations to estimate the indirect (herd) protection Tanzania | People 2 years and older Vaccine eligible (n=48178) Received the vaccine (n=23921) | None | Two doses of a killed whole-cell B-subunit cholera vaccine; None | Vaccine effectiveness of doses of cholera vaccine: 79% (95% CI 47, 92)  Reduced risk of cholera for people living in neighbourhoods of high vaccine coverage. Herd immunity was also found |
| 54 | Wierzba *et al.* [76] | Effectiveness of cholera vaccine for local staff, cold chain equipment staff and logistics staff to prevent clinically significant cholera in India | Patients, > 1 year, with acute diarrhoea Cases, cholera positive (n=44) | Patients, > 1 year, with acute diarrhoea, cholera negative (n=366) | Two (2) dose cholera vaccine; None | Vaccine effectiveness of 2 doses of cholera vaccine: 69.0% (95% CI 14.5, 88.8%) Single dose: 33%, test for trend, p = 0.0091 Incidence of cholera: 2.42/100 000 population |
| 55 | Franke *et al.* [77] | Effectiveness and duration of 2 doses and 1 dose of cholera vaccine | Participants ≥12 months, positive for Vibrio cholerae 01 (n=178) | 4 community controls selected for each case, from the same area (n=706) | Killed, bivalent, whole-cell oral cholera vaccine; None | Vaccine effectiveness of 2 doses of cholera vaccine over 4-years: 76% (95% CI 59, 86). Vaccine effectiveness of a single dose: 1^st^ year: 79% (95% CI 43, 93) Dropped zero by the end of 2nd year |
| 56 | Luquero *et al.* [75] | Effectiveness of the Shanchol vaccine in response to a cholera outbreak in an African country | Suspected cholera patients, > 12 years. Cases: cholera positive (n=40) | Neighbours, same age and sex as cases who did not seek care for diarrhoea during the outbreak (n=160) | Shanchol vaccine 2 dose cholera vaccine; None | Vaccine effectiveness with 2 complete doses: 86.6%; 95% CI 56.7, 95.8 (P = 0.001) |
